# Light-Driven Water Oxidation with Ligand-Engineered Prussian Blue Analogues

**DOI:** 10.1021/acs.inorgchem.1c03531

**Published:** 2022-02-24

**Authors:** Aliyu A. Ahmad, T. Gamze Ulusoy Ghobadi, Muhammed Buyuktemiz, Ekmel Ozbay, Yavuz Dede, Ferdi Karadas

**Affiliations:** † Department of Chemistry, Faculty of Science, Bilkent University, 06800 Ankara, Turkey; ‡ NANOTAM—Nanotechnology Research Center, Bilkent University, 06800 Ankara, Turkey; § Department of Chemistry, Faculty of Science, Gazi University Teknikokullar, 06500 Ankara, Turkey; ∥ Department of Electrical and Electronics Engineering, Bilkent University, 06800 Ankara, Turkey; ⊥ Department of Physics, Faculty of Science, Bilkent University, 06800 Ankara, Turkey; # UNAM—National Nanotechnology Research Center, Bilkent University, 06800 Ankara, Turkey

## Abstract

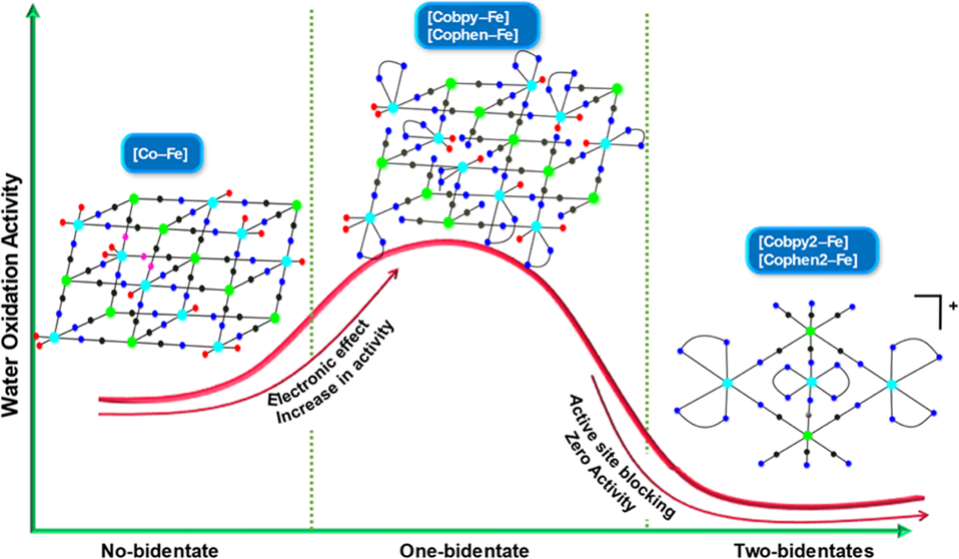

The elucidation of the ideal coordination environment of a catalytic site has been at the heart of catalytic applications. Herein, we show that the water oxidation activities of catalytic cobalt sites in a Prussian blue (PB) structure could be tuned systematically by decorating its coordination sphere with a combination of cyanide and bidentate pyridyl groups.  K_0.1_[Co(bpy)]_2.9_[Fe(CN)_6_]_2_ (**[Cobpy–Fe]**), K_0.2_[Co(phen)]_2.8_[Fe(CN)_6_]_2_ (**[Cophen–Fe]**), {[Co(bpy)_2_]_3_[Fe(CN)_6_]_2_}[Fe(CN)_6_]_1/3_ (**[Cobpy2–Fe]**), and {[Co(phen)_2_]_3_[Fe(CN)_6_]_2_}[Fe(CN)_6_]_1/3_ Cl_0.11_ (**[Cophen2–Fe]**) were prepared by introducing bidentate pyridyl groups (phen: 1,10-phenanthroline, bpy: 2,2′-bipyridine) to the common synthetic protocol of Co–Fe Prussian blue analogues. Characterization studies indicate that **[Cobpy2–Fe]** and **[Cophen2–Fe]** adopt a pentanuclear molecular structure, while **[Cobpy–Fe]** and **[Cophen–Fe]** could be described as cyanide-based coordination polymers with lower-dimensionality and less crystalline nature compared to the regular Co–Fe Prussian blue analogue (PBA), K_0.1_Co_2.9_[Fe(CN)_6_]_2_ (**[Co–Fe]**). Photocatalytic studies reveal that the activities of **[Cobpy–Fe]** and **[Cophen–Fe]** are significantly enhanced compared to those of **[Co–Fe]**, while molecular **[Cobpy2–Fe]** and **[Cophen2–Fe]** are inactive toward water oxidation. **[Cobpy–Fe]** and **[Cophen–Fe]** exhibit upper-bound turnover frequencies (TOFs) of 1.3 and 0.7 s^–1^, respectively, which are ∼50 times higher than that of **[Co–Fe]** (1.8 × 10^–2^ s^–1^). The complete inactivity of **[Cobpy2–Fe]** and **[Cophen2–Fe]** confirms the critical role of aqua coordination to the catalytic cobalt sites for oxygen evolution reaction (OER). Computational studies show that bidentate pyridyl groups enhance the susceptibility of the rate-determining Co(IV)-oxo species to the nucleophilic water attack during the critical O–O bond formation. This study opens a new route toward increasing the intrinsic water oxidation activity of the catalytic sites in PB coordination polymers.

## Abstract 2

Bidentate pyridyl groups are coordinated to the catalytic cobalt sites in a cyanide-based Co−Fe structure to afford well-tuned extended network structures, which exhibit an outstanding photocatalytic performance compared to the regular Co−Fe PBA.

## Introduction

Co–Fe Prussian blue analogues (PBAs) are three-dimensional (3D) coordination polymers with a general formula, A*_x_*Co*_y_*[Fe(CN)_6_]·*z*H_2_O, where A is an alkali or an alkaline metal ion positioned in the tetrahedral interstitial sites. It adopts a face-centered cubic (fcc) structure in the **Fm**3*m* space group. Iron and cobalt sites are connected through cyanide bridging ligands to afford an extended framework.^1^ Co–Fe PBAs have been attractive compounds for heterogeneous water oxidation due to their facile synthesis procedures, stabilities, fast electron transfer between Fe to Co sites through the short cyanide bridge, and easily tunable metal sites.^2^ Following the leading study by Galán-Mascarós and co-workers in 2013,^3^ several research endeavors have been devoted to elucidating the origin of the activity and to enhancing the catalytic performance of Co–Fe PBAs.^4−6^ These studies indicate that the cobalt sites with accessible sites, which are on the surface or in the vacancies created to provide charge balance, are the catalytic active sites for water oxidation. Although the iron site does not serve as a catalytic site due to the lack of an accessible coordination site, it plays an important electronic role in enhancing the activity and the stability of cobalt sites.

Studies in this field could be divided into four main strategies. (i) Utilizing the PBA as a precatalyst: the majority of the studies focus on decomposing the cyanide network to their corresponding oxides or phosphides or selenides to form what is referred to as “PB derived catalyst”. For instance, Zhang et al. converted Co–Fe PBA to (Fe–Co)Se_2_ by a selenization procedure. The derived catalyst with high porosity and fast electron transfer exhibits superior activity and long-term stability.^7^ (ii) Coupling PBAs with a light-absorbing component: a strategy commonly employed in this field is to couple PBA with proper semiconductor (SC) or photosensitizers.^8,9^ This strategy aims to achieve a proper energy level matching between the valence band of SC and the highest occupied molecular orbital (HOMO) of the catalytic site to boost the oxygen evolution reaction (OER) activity and the stability of PB-based assembly. Several Co–Fe PBA catalyst/visible-light-absorbing SC assemblies have been designed by our group, e.g., a Co–Fe PBA catalyst was coupled with layered double hydroxide (LDH),^10^ BiVO_4_,^11^ niobates,^12^ and brown-TiO_2_^9^ in different studies, in which each of these assemblies displayed an excellent increase in activity and stability. In addition to our work, Shi et al. also designed an anisotropic PBA–TiO_2_ Janus nanoreactor that displayed improved photocatalytic activity compared to ordinary PBA or TiO_2_.^13^ Furthermore, Co–Fe PBA/CoS_2_ hybrid prepared by Xu et al. gave rise to a remarkable increase in activity due to the efficient electron transfer between CoS_2_ and Co–Fe PBA.^14^ (iii) Changing the cyanoiron precursor: another adopted strategy is to chemically modify or tune the iron site, which consequently has an indirect electronic effect on the activity of the cobalt sites.^4^ For instance, our group reported that replacing the hexacyanoferrate precursor with a polymer-bound pentacyanoferrate results in the formation of an amorphous structure with an enhanced activity.^15^ Pires et al. leveraged on comparative studies to understand the influence of ligands attached to the Fe sites of Co–Fe PBA on its photoactivity.^16^ (iv) Changing the cobalt site: few studies on directly tuning the catalytic cobalt sites without decomposition in the cyanide network are focused on changing the cobalt metal entirely to different metal, partially substituting, or doping with a different metal.^17−19^

Despite the growing research in this field and all of the efforts to increase the activity of cobalt sites, direct tuning of the coordination sphere of catalytic cobalt sites in Co–Fe PBAs still remains uncharted territory. Herein, we report a series of Co–Fe PBAs, which consist of cobalt sites surrounded by a combination of bidentate pyridyl ligands, cyanide groups, and water molecules. The facile synthetic method allows the tuning of the number and the type of bidentate pyridyl ligands coordinated to the cobalt site in a PB network structure, which provides an ideal platform to elucidate the structure and activity relationship in PBAs. We prepared and characterized a series of 3D (**[Co–Fe]**), low-dimensional (**[Cobpy–Fe]**, **[Cophen–Fe]**), and molecular (**[Cobpy2–Fe]**, **[Cophen2–Fe]**) cyanide coordination compounds, simply by decorating the coordination sphere of the cobalt sites with zero, one, and two bidentate pyridyl ligands (phen and bpy), respectively. The compounds were characterized by infrared, X-ray diffraction (XRD), X-ray photoelectron spectroscopy (XPS), and electrochemical techniques, and their activities were evaluated with photocatalytic experiments. Furthermore, the origin of enhanced catalytic activities in **[Cobpy–Fe]** and **[Cophen–Fe]** was elucidated by electronic structure calculations.

## Experimental Section

### Chemicals and Reagents

All reagents and solvents used were of high analytical grade and used without any further purifications. Milli Q deionized water (resistivity: 18 MΩ·cm) was used to prepare all of the solutions.

#### Cobalt Precursor Synthesis

##### Synthesis of Mono(2,2′-bipyridine)dichlorocobalt(II), CobpyCl_2_

Two millimoles of 2,2′-dipyridyl in 20 mL of acetone were mixed with a solution containing 2 mmol of anhydrous cobalt(II) chloride in 20 mL of acetone. The resulting solution was stirred for 2 h, filtered, and dried in a desiccator to obtain a light-blue precipitate. Yield: 486.5 mg (85%). Anal. calcd (%) for C_10_H_8_N_2_Cl_2_Co: C, 41.94; H, 2.78; N, 9.79. Found: C, 43.27; H, 2.86; N, 9.96.

##### Synthesis of Mono(1,10-phenanthroline)dichlorocobalt(II), CophenCl_2_

Two millimoles of 1,10-phenanthroline monohydrate in 20 mL of acetone were mixed with a solution containing 2 mmol of anhydrous cobalt(II) chloride in 20 mL of acetone. The resulting solution was stirred for 2 h, filtered, and dried in a desiccator to obtain a blue precipitate. Yield: 600.8 mg (85%). Anal. calcd (%) for C_12_H_8_N_2_Cl_2_Co: C, 46.43; H, 2.58; N, 9.02. Found: C, 47.22; H, 2.85; N, 8.79.

##### Synthesis of Bis(2,2′-bipyridine)dichlorocobalt(II), Cobpy_2_Cl_2_

Four millimoles of 2,2′-dipyridyl in 20 mL of acetone were mixed with a solution containing 2 mmol of anhydrous cobalt(II) chloride in 20 mL of acetone. The resulting solution was stirred for 2 h, filtered, and dried in a desiccator to obtain an orange-red precipitate. Yield: 817 mg (90%). Anal. calcd (%) for C_20_H_16_N_4_Cl_2_Co: C, 54.26; H, 3.62; N, 12.66. Found: C, 53.50; H, 3.52; N, 12.45.

##### Synthesis of Bis(1,10-phenanthroline)dichlorocobalt(II), Cophen_2_Cl_2_

Four millimoles of 1,10-phenanthroline monohydrate in 20 mL of acetone were mixed with a solution containing 2 mmol of anhydrous cobalt(II) chloride in 20 mL of acetone. The resulting solution was stirred for 2 h, filtered, and dried in a desiccator to obtain a red precipitate. Yield: 1065.5 mg (90%). Anal. calcd (%) for C_24_H_16_N_4_Cl_2_Co: C, 58.73; H, 3.26; N, 11.42. Found: C, 57.88; H, 3.19; N, 11.19.

#### Catalyst Synthesis

##### K_0.1_Co_2.9_[Fe(CN)_6_]_2_·12H_2_O, [Co–Fe]

**[Co–Fe]** was synthesized according to a previously reported method with slight changes.^20^ Briefly, a 50 mL aqueous solution of 0.75 mmol of Co(NO_3_)_2_·6H_2_O was added dropwise to an equal volume of an aqueous solution of 0.5 mmol of K_3_Fe(CN)_6_ under constant stirring. The resulting solution was stirred vigorously for 2 h, centrifuged, washed with deionized water, and dried in the oven at 60 °C to obtain a brown powder. Yield: 267 mg (66%). Anal. calcd (%) for C_12_H_22_N_12_O_12_K_0.1_Co_2.9_Fe_2_: C, 17.69; H, 2.94; N, 20.61. Found: C, 17.19; H, 2.84; N, 19.52. EDX Co/Fe atomic ratio: 3:2.

All of the remaining cyanide-bridged compounds used as catalysts were prepared using a similar method. Therefore, only the synthesis of **[Cobpy–Fe]** is discussed in detail.

##### K_0.1_[Co(bpy)]_2.9_[Fe(CN)_6_]_2_·7.5H_2_O, [Cobpy–Fe]

Twenty milliliters of an aqueous solution of CobpyCl_2_ (0.6 mmol) was added to 20 mL of an aqueous solution of K_3_Fe(CN)_6_ (0.4 mmol). The resulting solution was stirred for 1 h and allowed to stand overnight. Then, the obtained green precipitate was centrifuged, washed, and dried in an oven at 50°C for approximately 20 h. Yield: 285 mg (60%). Anal. calcd (%) for C_41_H_38.2_N_17.8_O_7.5_K_0.1_Co_2.9_Fe_2_: C, 41.45; H, 3.21; N, 20.99. Found: C, 41.84; H, 2.85; N, 20.16. EDX Co/Fe atomic ratio: 3:2.

##### K_0.2_[Co(phen)]_2.8_[Fe(CN)_6_]_2_·7.5H_2_O, [Cophen–Fe]

CophenCl_2_ was used as a cobalt precursor and a similar procedure as that used for **[Cobpy–Fe]** was followed to obtain a green precipitate. Yield: 256 mg (52%). Anal. calcd (%) for C_42_H_37.4_N_17.6_ O_7.5_K_0.2_Co_2.8_Fe_2_: C, 44.25; H, 3.02; N, 19.92. Found: C, 43.17; H, 2.80; N, 18.98. EDX Co/Fe atomic ratio: 3:2.

##### {[Co(bpy)_2_]_3_[Fe(CN)_6_]_2_}[Fe(CN)_6_]_1/3_·14.5H_2_O, [Cobpy2–Fe]

Cobpy_2_Cl_2_ was used as a cobalt precursor and a similar procedure as that used for **[Cobpy–Fe]** was followed to obtain a blue precipitate. Yield: 348 mg (55%). Anal. calcd (%) for C_74_H_53_N_26_O_29_Co_3_Fe_2.3_: C, 47.49; H, 4.06; N, 19.47. Found: C, 46.08; H, 3.72; N, 18.53. EDX Co/Fe atomic ratio: 3:2.4.

##### {[Co(phen)_2_]_3_[Fe(CN)_6_]_2_}[Fe(CN)_6_]_1/3_Cl_0.11_·17.5H_2_O, [Cophen2–Fe]

Cophen_2_Cl_2_ was used as the cobalt precursor and a similar procedure as that used for **[Cobpy–Fe]** was followed to obtain a light-blue precipitate. Yield: 413 mg (59%). Anal. calcd (%) for C_86_H_59_N_26_O_35_Cl_0.11_Co_3_Fe_2.3_: C, 49.81; H, 4.01; N, 17.57. Found: C, 47.58; H, 3.23; N, 17.28. EDX Co/Fe atomic ratio: 3:2.3.

### Physical Measurements

The surface morphology of the catalyst was revealed from scanning electron microscopy (SEM) images (FEI QUANTA 200 FEG ESEM), the instrument is equipped with an Ametek EDAX energy-dispersive X-ray (EDX) system for elemental composition analysis. The powder X-ray diffraction (PXRD) patterns were obtained using a PANalytical X’pert PRO X-ray diffractometer using Cu Kα radiation (1.5406 Å). Infrared (IR) spectra were recorded on a Bruker α Platinum-ATR spectrometer within the wavenumber range of 400–4000 cm^–1^ for 64 scans. X-ray photoelectron spectroscopy (XPS) analysis was performed on a Thermo Fisher Scientific K-Alpha X-ray photoelectron spectrometer using an Al Kα microfocused monochromator as the X-ray source and a flood gun for charge neutralization. All of the peaks were shifted with reference to the C 1s peak position (284.8 eV). UV–vis absorption spectra of the cobalt precursors were obtained on Agilent Cary 5000 UV–vis–NIR spectrophotometer using a quartz cuvette with a path length of 1 cm. Thermogravimetric analysis (TGA) was carried out on a Q500 thermogravimetric analyzer within the temperature range of 30–800 °C at 5 °C/min under a nitrogen atmosphere. CHN elemental analysis was obtained on a Thermo Scientific FLASH 2000 Series CHNS/O elemental analyzer using BBOT as a standard and V_2_O_5_ as a catalyst.

### Photocatalytic OER Experiment

Photocatalytic experiments were conducted in a Pyrex flask totally sealed with a septum. Thirty milliliters of a 0.1 M potassium phosphate buffer solution (PBS) containing 10 mg of catalyst, 5 mM sodium persulfate (Na_2_S_2_O_8_), and 1 mM ruthenium photosensitizer ([Ru(bpy)_3_]Cl_2_) was prepared. PBS was prepared by mixing aqueous solutions of KH_2_PO_4_ (0.1 M) and K_2_HPO_4_ (0.1 M). The Pyrex flask was covered with aluminum foil before adding the ruthenium complex to prevent an early light-induced reaction. Initially, the mixture was purged with N_2_ gas thoroughly for 25–30 min. The photocatalytic experiment was carried out for 1 h, and the amount of oxygen evolved was determined by injecting 100 μL of the headspace gas at a 15 min interval into a gas chromatograph (Agilent 7820A, a gas chromatograph equipped with a molecular sieve and a thermal conductivity detector (TCD), using argon as the carrier gas). The experiment was conducted at least twice for each catalyst to obtain a reproducible result.

### Electrochemical Experiments

Electrochemical experiments were conducted on a Gamry Instruments Interface 1000 potentiostat/galvanostat at 25 °C. Using the conventional three-electrode setup, with Pt mesh as the counter electrode, Ag/AgCl (3.5 M KCl) as the reference electrode, and fluorine-doped tin oxide (FTO) coated electrode (∼80% transmittance; 2 mm slides with 7 Ω·sq^–1^ surface resistivity and 1 cm × 2 cm size) as the substrate for the working electrode. Cyclic voltammetry experiments were performed in a PBS at pH 7 containing 1 M KNO_3_ as the supporting electrolyte.

### Working Electrode Preparation

A 1 cm × 2 cm FTO electrode was used as the working electrode, but only 1 cm × 1 cm of the conducting surface was coated with the catalyst. Prior to coating the FTO surface, the electrode was adequately cleaned by sonicating for 10 min in a basic soapy solution, deionized water, and isopropanol and then annealed at 350 °C in the furnace. The surface of FTO was coated with the catalyst by following a previously reported two-step in situ method with slight changes.^15^ Briefly, 1000 μL of a 0.05 M aqueous solution of hexacyanoferrate was spin-coated on FTO at 500 rpm for 150 s, air-dried, and dipped into a 0.075 M aqueous solution of Co^2+^ precursor for 15 min. This procedure was repeated three times. Finally, the electrode was dried in an oven at 60 °C for about 10 min, washed with deionized water, air-dried, and kept in the desiccator until used.

## Results and Discussion

### Synthesis and Characterization of Cobalt Precursors

The cobalt complex precursors were prepared following the previously established protocols^21,22^ by simply mixing a proper equivalent of bpy or phen ligands with cobalt ions to afford mononuclear cobalt bipyridyl complexes. One equivalent of bidentate pyridyl ligand was used to obtain CobpyCl_2_ and CophenCl_2_, while 2 equiv were used to obtain Cobpy_2_Cl_2_ and Cophen_2_Cl_2_. A color change was observed with naked eyes as the precursors were mixed. The UV–vis spectra of the complexes display an MLCT band (∼475 nm) in the visible region, which originates from the electron transfer from the d-orbital of the Co to the π*-orbital of the ligands (Figure S1). The remaining bands below 350 nm are attributed to π–π* and n−π* transitions of the ligands.^23^ The similarity in the visible region of the absorption spectra (Figure S1 inset) of CobpyCl_2_ and CophenCl_2_ proves that cobalt complex with one pyridyl ligand is successfully synthesized. A similar trend is obtained for Cobpy_2_Cl_2_ and Cophen_2_Cl_2_. The Fourier transform infrared (FTIR) technique was also used to verify the formation of these complexes (Figure S2). The bands that were obtained match well with the fingerprints of the bpy and phen ligands, with a slight shift indicating the formation of the cobalt complex. The sharp band at ∼1600 cm^–1^, the one at around 1200 cm^–1^, the broad bands at ∼750–1000 cm^–1^, and the weak bands at 2900–3100 cm^–1^ correspond to C=N/C=C stretches, C–C/C–N bending, aromatic C–H vibrations, and C–H stretching frequencies, respectively.^24^

### Synthesis and Characterization

**Scheme 1:**
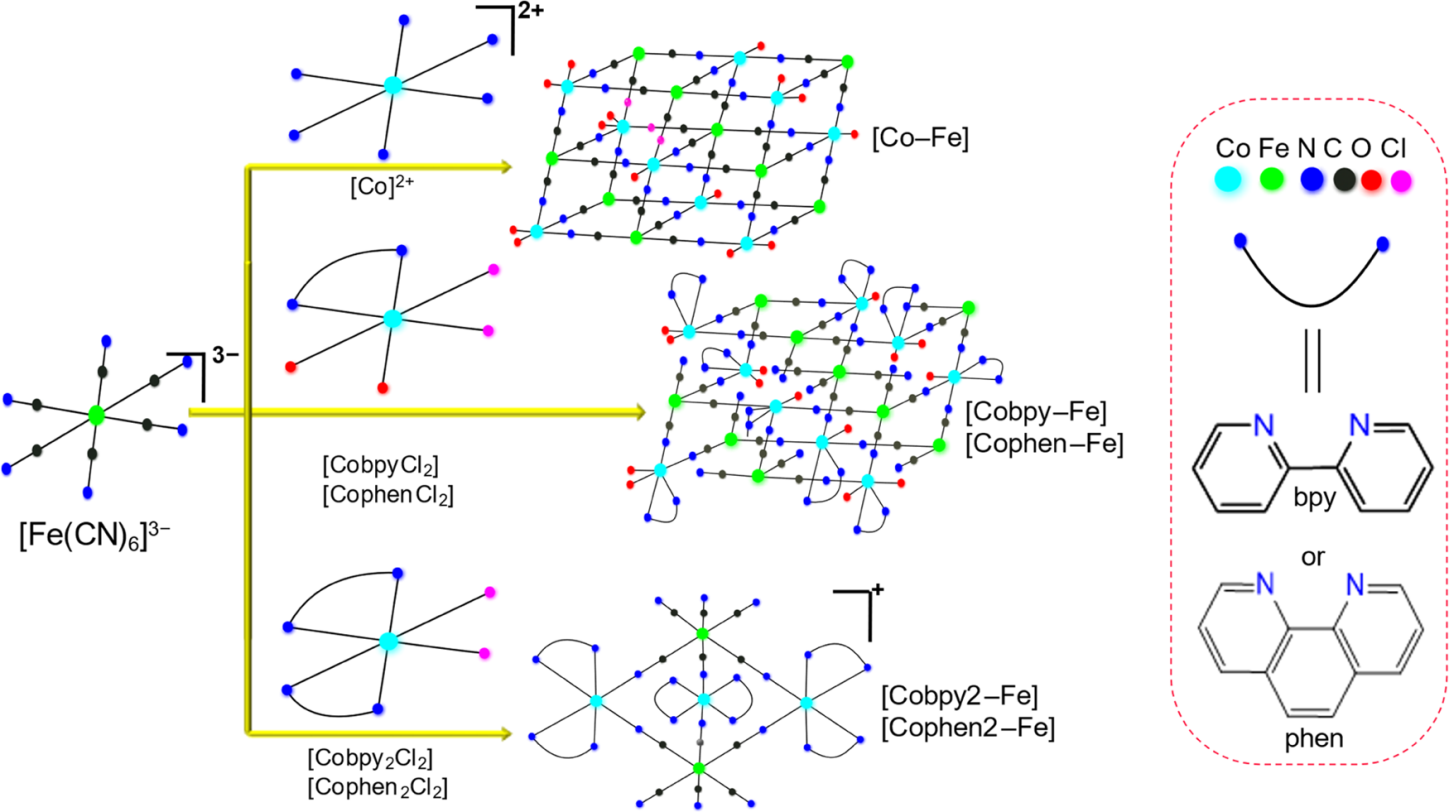
Schematic Illustration for the Synthesis and Structural Units of Compounds: All Five Compounds Are Derived from the Same Precursor, K_3_Fe(CN)_6_ The resulting cyanide coordination compounds differ in their Co precursors; (top) 3D crystalline **[Co–Fe]**, (middle) low-dimensional **[Cobpy–Fe]** and **[Cophen–Fe]**, and (bottom) molecular **[Cobpy2–Fe]** and **[Cophen2–Fe]** compounds. The curved lines represent bpy and phen ligands framed by blue circles.

All compounds **[Co–Fe]**, **[Cobpy–Fe]**, **[Cophen–Fe]**, **[Cobpy2–Fe]**, and **[Cophen2–Fe]** were synthesized via a co-precipitation technique, which involves simply mixing 3 equiv of the Co^2+^ precursor with 2 equiv of hexacyanoferrate complex (see Scheme 1). SEM images at a 10 μm magnification (Figure S3) reveal that **[Co–Fe]** exhibits a different surface morphology compared to the other compounds. **[Co–Fe]** shows aggregated semi-cube-like crystals, in agreement with XRD patterns, while **[Cobpy–Fe]** and **[Cophen–Fe]** exhibit amorphous nature with a rough surface due to the contraction of the lattice parameter. **[Cobpy2–Fe]** and **[Cophen2–Fe]** images display agglomeration into clusters. The chemical formula of the compounds was estimated based on EDX elemental composition analysis, CHN elemental analysis, and thermogravimetric analysis (TGA) (Tables S1 and S2). A Co/Fe atomic ratio of 3:2 is obtained by EDX analysis. The number of water molecules present in each compound was ascertained from thermogravimetric analysis (TGA) (Figure S4). The gradual decrease in TGA curves ranging from 30 to 150 °C is attributed to the loss of coordinated and non-coordinated water molecules, while the steep decrease in temperature above 250 °C corresponds to the decomposition/transformation of the cyanide network to an oxide.^25^

**Figure 1:**
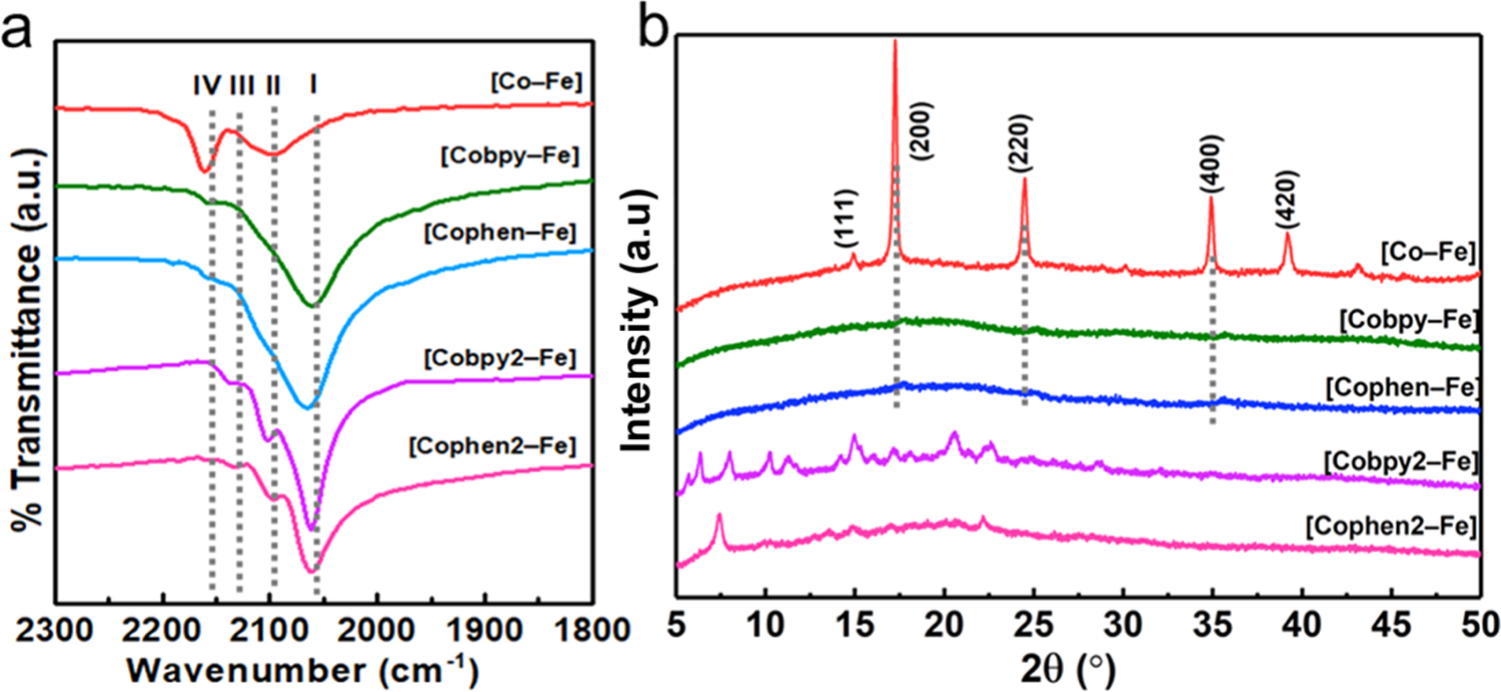
(a) ATR-FTIR spectra showing the cyanide stretching region of the compounds with four different assignments: (I) terminal Fe^2+^–CN, (II) terminal Fe^3+^–CN and bridging Fe^2+^–CN–Co^2+^, (III) bridging Fe^2+^–CN–Co^3+^, and (IV) bridging Fe^3+^–CN–Co^2+^ coordination mode. (b) PXRD patterns of compounds.

FTIR technique is employed mainly to investigate the nature of the cyanide bond and the presence of pyridyl groups in these compounds. The presence of pyridyl bands in the fingerprint region of the FTIR spectra clearly distinguishes **[Cobpy–Fe]**, **[Cophen–Fe]**, **[Cobpy2–Fe]**, and **[Cophen2–Fe]** from **[Co–Fe]** (Figure S5). The characteristic cyanide stretching vibration (ν(CN)) is observed for all of the compounds in the 2000–2200 cm^–1^ region (Figure 1a). **[Co–Fe]** exhibits a strong band at 2162 cm^–1^ with a medium one at 2098 cm^–1^, attributed to the Fe^3+^–CN–Co^2+^ and Fe^2+^–CN–Co^2+^ coordination modes, respectively.^26^ The partial reduction in the oxidation state of Fe from 3+ to 2+ and vice versa for the cobalt site indicates the formation of mixed-metal compounds due to the electron transfer between the metal sites, which is a common phenomenon called metal-to-metal charge transfer in Co–Fe PBAs.^27^**[Cobpy–Fe]** and **[Cophen–Fe]** display a similar broad ν(CN) band extending from 2020 cm^–1^ to 2132 cm^–1^, which are assigned to the Fe^2+^–CN–Co^3+^ bridging mode and the Fe^2+^–CN terminal coordination, the shoulder ν(CN) band at 2156 cm^–1^, is attributed to Fe^3+^–CN–Co^2+^ bridging mode. The presence of the terminal Fe^2+^–CN coordination mode could be attributed to the disorder of the extended PBA structure due to the coordination of pyridyl ligands to the cobalt site. The IR spectra of **[Cobpy2–Fe]** and **[Cophen2–Fe]** also exhibit a broad ν(CN) band at 2063 cm^–1^ and a shoulder ν(CN) one at 2135 cm^–1^, which are attributed to Fe^2+^–CN terminal and Fe^2+^–CN–Co^3+^ bridging modes, respectively. The medium ν(CN) band at 2098 cm^–1^ is attributed to Fe^3+^–CN terminal coordination, which belongs to the [Fe(CN)_6_]^3–^ counter anion. In all Co(pyridyl)–Fe compounds, the Co^3+^ sites dominate over the Co^2+^ ones since the low-spin d^6^ state is preferred over the high-spin d^7^ case.^28^ This high oxidation state also favors the formation of high-valent Co(IV)-oxo species required for water oxidation.^29^

The crystal structures of the synthesized compounds were analyzed using PXRD (Figure 1b). As expected, **[Co–Fe]** adopts a face-centered cubic (fcc) structure with diffraction peaks at 14.77, 17.30, 24.56, 35.02, and 39.30° allotted to the (111), (200), (220), (400), and (420) reflection planes, respectively.^30^ The PXRD patterns of **[Cobpy–Fe]** and **[Cophen–Fe]** exhibit three main peaks at 17.63, 25.10, and 35.91, which are attributed to the (200), (220), and (400) reflection planes of cubic PB network similar to **[Co–Fe]**. While these characteristic peaks confirm the formation of PB cubic structure for **[Cobpy–Fe]** and **[Cophen–Fe]**, the broadening and the weak intensities of the peaks suggest a decrease in the dimensionality of the extended structure (Figure 1b). Diffraction peaks also shift to higher angles compared to **[Co–Fe]**, indicating a slight shortening of the cubic cell parameter (**[Co–Fe]***a* = 10.243 Å; **[Cobpy–Fe]***a* = 10.011 Å; **[Cobpy–Fe]***a* = 10.051 Å).^31^ The PXRD peaks of **[Cobpy2–Fe]** align perfectly with a reference molecular compound previously reported by Berlinguette et al. (Figure S6a).^32^**[Cobpy2–Fe]** consists of a {[Co(bpy)_2_]_3_[Fe(CN)_6_]_2_}^+^ cation and a [Fe(CN)_6_]^3–^ counter anion. Although **[Cophen2–Fe]** and **[Cobpy2–Fe]** are trigonal bipyramidal compounds (Figure S6b,c),^33^ the position of the diffraction peaks are slightly altered and are broader in **[Cophen2–Fe]** when compared to **[Cobpy2–Fe]**, which could be attributed to the difference in the size of the ligands and possibly the type of counterions. Therefore, PXRD, FTIR, CHN elemental analysis, and EDX analysis suggest that **[Cobpy2–Fe]** and **[Cophen2–Fe]** are isostructural. This claim is further supported by XPS analysis.

**Figure 2:**
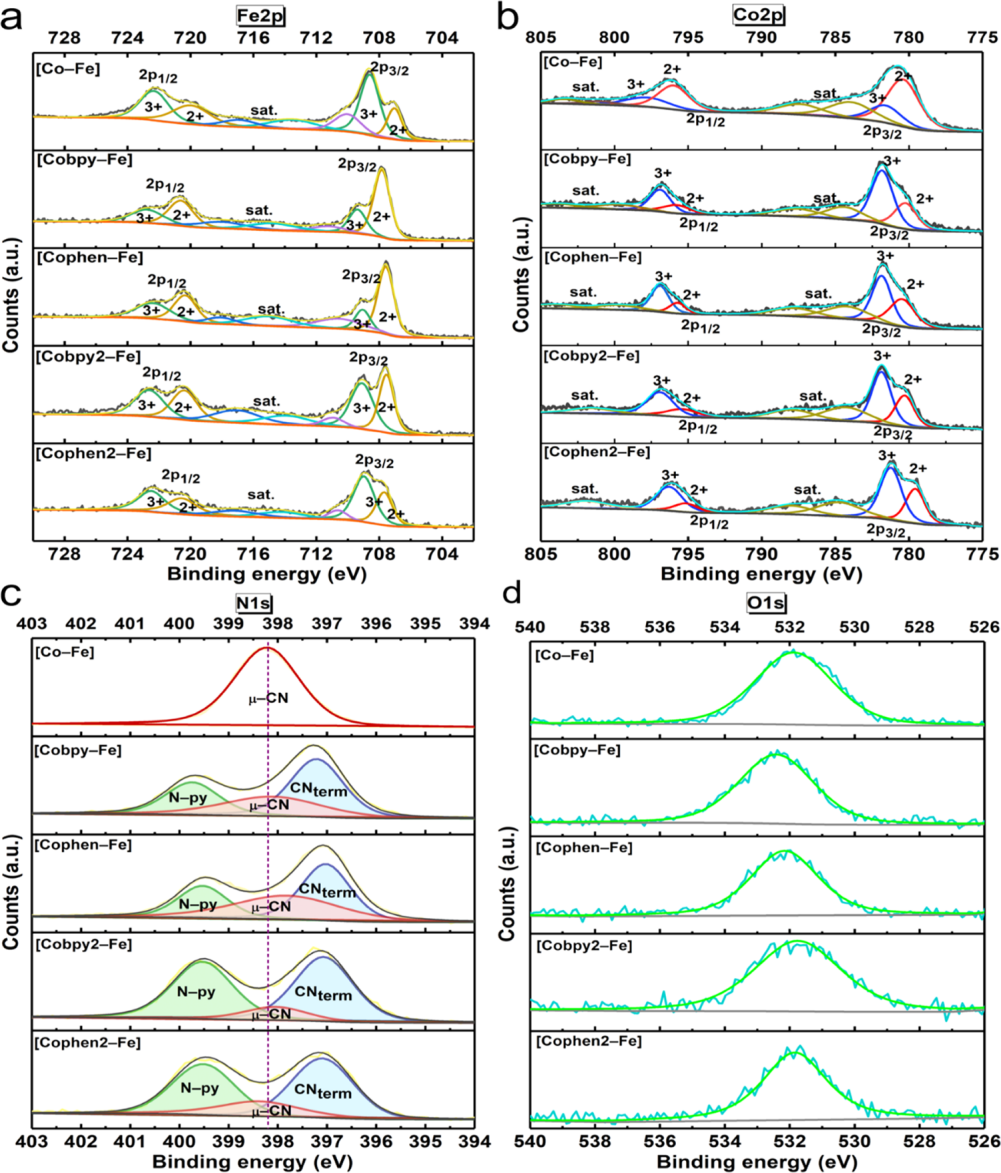
High-resolution XPS spectra of (a) Fe 2p, (b) Co 2p, (c) N 1s, and (d) O 1s peaks for the obtained samples.

The structure of the PB compounds is further elucidated with XPS analysis. For all of the compounds, the Fe 2p signal (∼704–728 eV) is deconvoluted into Fe 2p_3/2_ and Fe 2p_1/2_ peaks, each fitted into Fe^2+^ and Fe^3+^ peaks (Figure 2a). Similarly, the Co 2p signal (∼775–805 eV) is deconvoluted into Co 2p_3/2_ and Co 2p_1/2_ peaks, each of which is fitted into Co^2+^ and Co^3+^ peaks (Figure 2b).^34,35^ A shake-up satellite peak is observed between the 3/2 and 1/2 spin states of both Fe 2p and Co 2p signals. In **[Co–Fe]**, a higher atomic percentage of Co^2+^ to Co^3+^ ions is observed, suggesting that Fe^3+^–CN–Co^2+^ is the dominant coordination mode. However, for all of the Co(pyridyl)–Fe compounds, a higher atomic percent of Co^3+^ ions is obtained than Co^2+^ ions. Therefore, Fe^2+^–CN–Co^3+^ is the dominant coordination mode when cobalt sites are surrounded by electron-accepting bidentate pyridyl ligands. This result is also consistent with the cyanide stretches observed in the FTIR spectra. As displayed in Figure 2c, the N 1s signal of **[Co–Fe]** reveals one peak assigned to the bridging-CN (μ-CN). On the other hand, the N 1s regions of Co(pyridyl)–Fe compounds are fitted into three peaks at 396.43, 397.9, and 398.95 eV, which are attributed to the terminal-CN (CN_term_), bridging-CN (μ-CN), and the pyridyl-N (N-py), respectively. Furthermore, the atomic ratio of the pyridyl-N peak in **[Cobpy2–Fe]** is approximately 2 times higher than that of **[Cobpy–Fe]**, suggesting that the bipyridyl groups coordinated to the cobalt sites are retained in the Co–Fe compounds. As expected, a similar trend is observed for **[Cophen2–Fe]** to **[Cophen–Fe]**. The O 1s signal observed at 532.4 eV for all compounds is assigned to the coordinated and non-coordinated water molecules (Figure 2d). A peak in the 529–530 eV region due to a possible metal oxide formation is not observed, which rules out the transformation of the structure to oxides during the synthesis.^11,36^ The C 1s core-level XPS signal at 284.8 eV is present as well in all of the samples (Figure S7).

### Photocatalytic Studies

**Figure 3:**
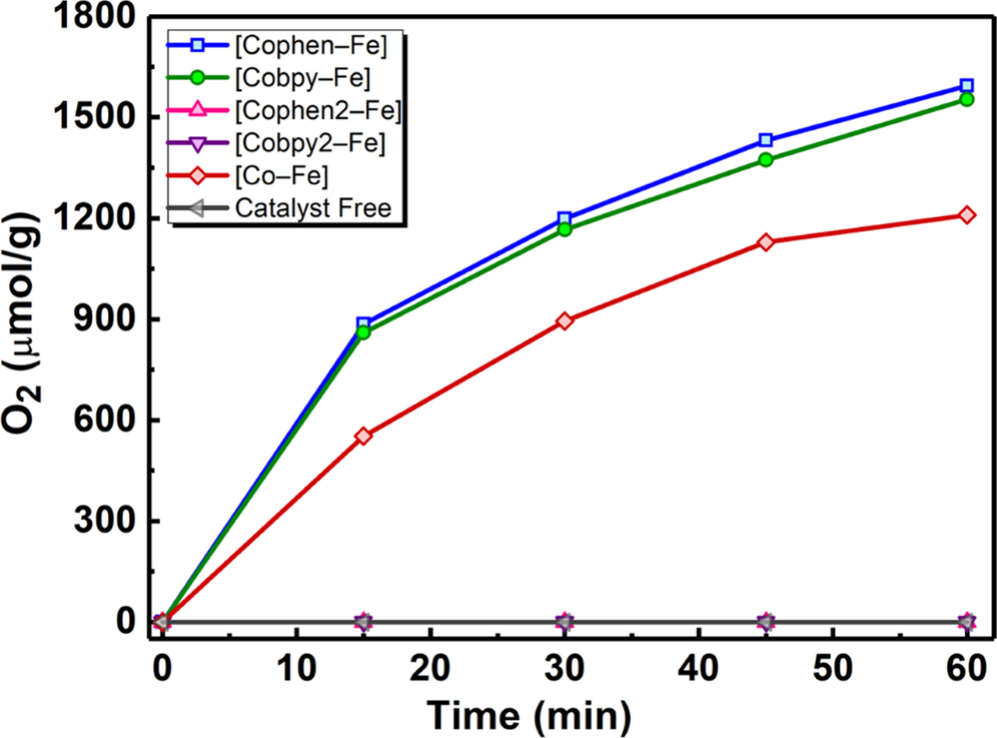
Photocatalytic oxygen evolution activities of the compounds in a 0.1 M PBS (pH 7) containing 10 mg of catalyst, 1 mM [Ru(bpy)_3_]^2+^ photosensitizer, and 5 mM Na_2_S_2_O_8_ as the sacrificial electron scavenger with a white light source, 100 mW·cm^–2^.

The photocatalytic water oxidation experiments were performed in a PBS solution, using [Ru(bpy)_3_]Cl_2_ as the photosensitizer and sodium persulfate as the electron scavenger. During a 1 h photocatalytic experiment, **[Cophen–Fe]** and **[Cobpy–Fe]** exhibit the highest activities of 1594 and 1553 μmol g^–1^ h^–1^, respectively, while **[Co–Fe]** reaches an activity of 1210 μmol g^–1^ h^–1^ (Figure 3). An approximately 30% increase in the catalytic activity could be attributed to the tuning of electron density of catalytic cobalt sites in **[Cophen–Fe]** and **[Cobpy–Fe]** with electron-withdrawing bidentate pyridyl groups, which will be discussed in the following sections in detail.

Furthermore, **[Cobpy2–Fe]** and **[Cophen2–Fe]** show no oxygen evolution due to the lack of catalytic sites since all of the cobalt sites in these pentanuclear molecular complexes are fully coordinated to six −NC groups, thereby preventing an aqua (water) coordination to the cobalt sites. The complete inactivity of these complexes, therefore, signifies that the metal sites in cyanide-based Co–Fe compounds do not release the pyridyl or cyanide groups under photocatalytic conditions.

### Postcatalytic Characterization

**Figure 4:**
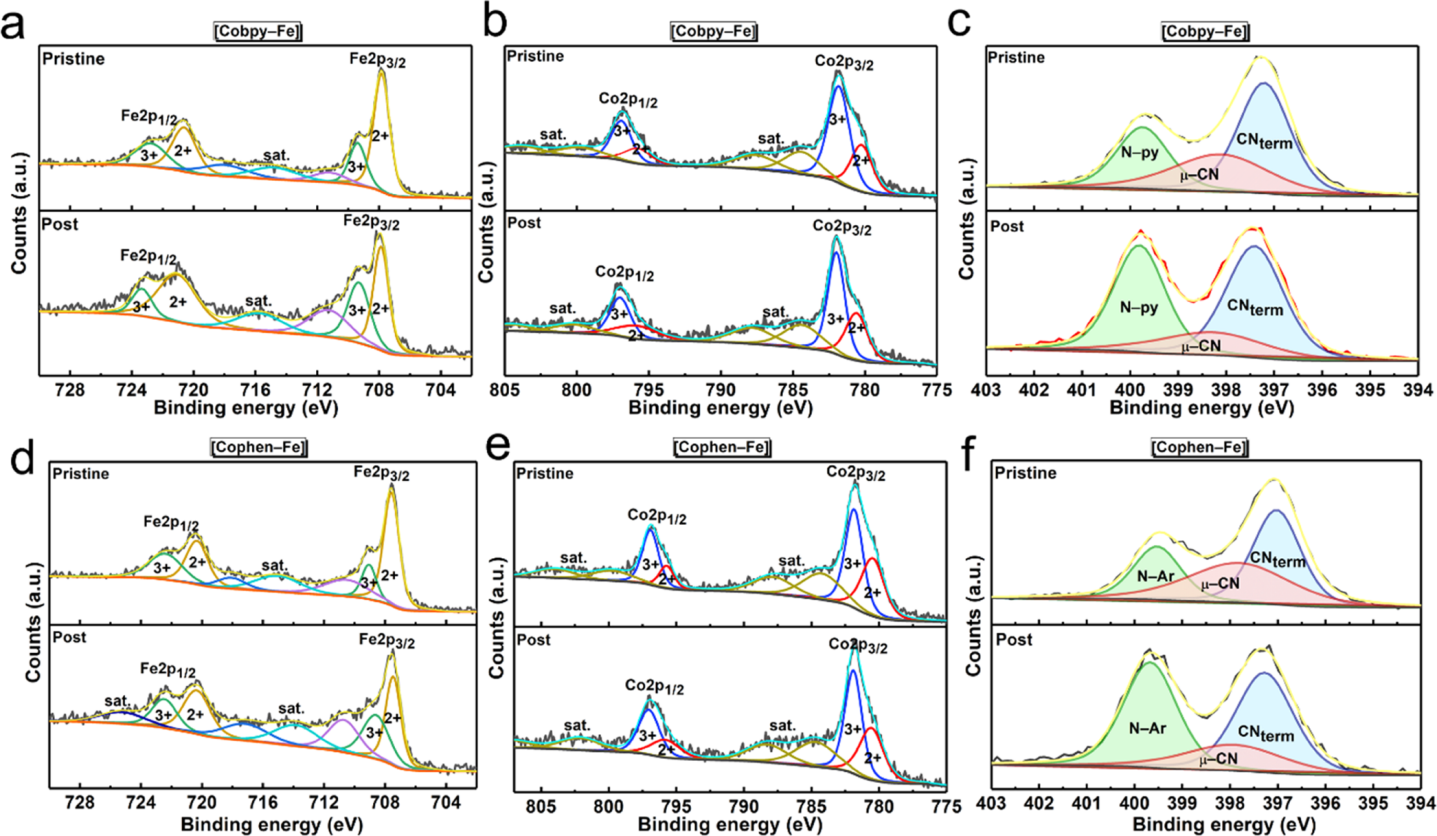
High-resolution XPS spectra of (a, d) Fe 2p, (b, e) Co 2p, and (c, f) N 1s from the pristine and postcatalytic samples of **[Cobpy–Fe]** and **[Cophen–Fe]**, respectively.

Postcatalytic FTIR and XPS studies were performed further to confirm the stability of the catalysts under photocatalytic conditions. No distinguishable differences between the O 1s XPS signals of the pristine and postcatalytic powder samples of **[Cobpy–Fe]** and **[Cophen–Fe]** are observed (Figure S8b,d). Moreover, the absence of a lattice cobalt oxide peak in the 529–530 eV region of the postcatalytic samples rules out the possible transformation of the cyanide-based compounds to metal oxides.^11^ The only present peak at ∼532.4 eV is assigned to the coordinated and non-coordinated water molecules in the compound. Similar Fe 2p and Co 2p XPS peaks are obtained in the pristine and postcatalytic XPS spectra (Figure 4a,b,d,e), suggesting that the metal centers are intact.

**Figure 5:**
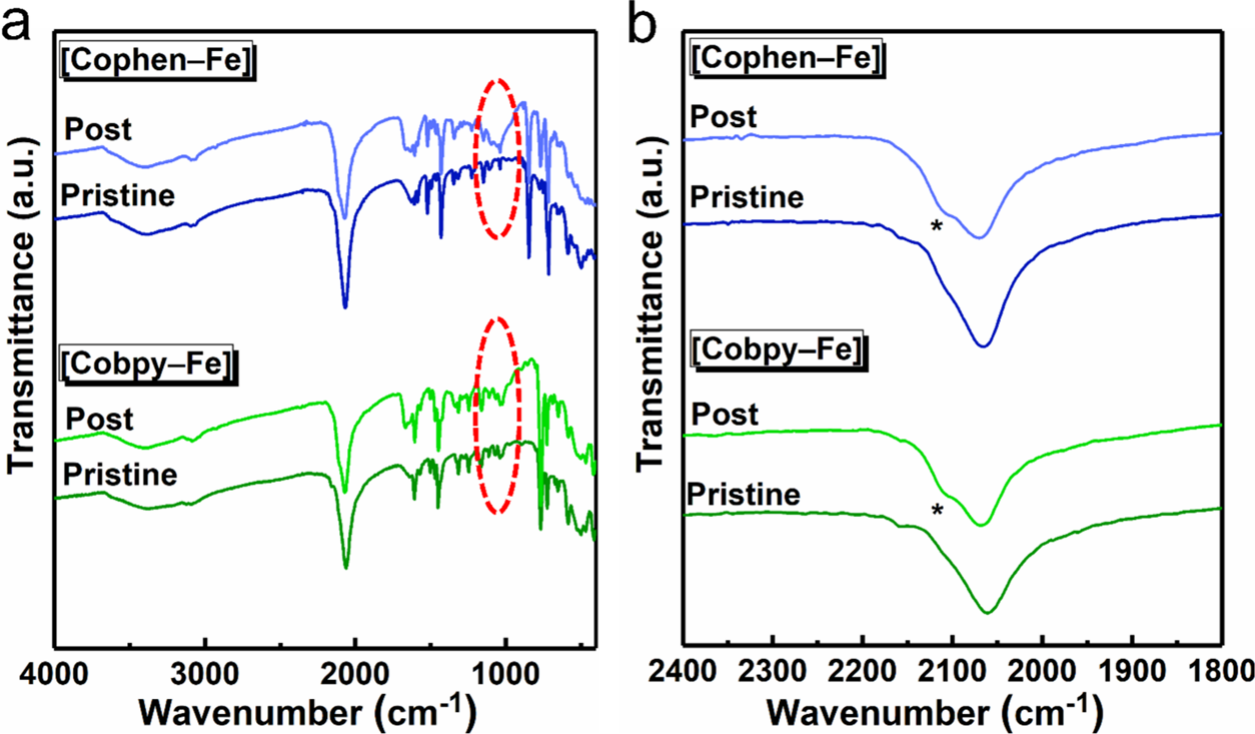
FTIR spectra of the pristine and postcatalytic samples of [Cobpy–Fe] and [Cophen–Fe] showing (a) the full spectrum ranging from 4000 to 400 cm^–1^ (the encircled region from 900 to 1100 cm^–1^ indicates the coordination of bipyridyl ligands to the Co–Fe structures) and (b) cyanide stretching region ranging from 2400 to 1800 cm^–1^ (the asterisk point at ∼2100 cm^–1^ is ascribed to structural changes in the cyanide environment due to the coordination of bipyridyl groups to cobalt sites).

The postcatalytic FTIR analysis reveals an additional small band in the cyanide region (∼2100 cm^–1^) and a broadening around 900–1100 cm^–1^ (Figure 5a,b) compared to the spectra of the pristine samples. Both changes could be attributed to a phenomenon referred to as bipyridyl poisoning of the catalyst.^6^ In a photocatalytic process in the presence of [Ru(bpy)_3_]^2+^/S_2_O_8_^2–^ couple, the ruthenium complex decomposes by releasing bipyridyl groups. The bpy groups could then coordinate to the catalytic sites and inactivate them for water oxidation. The appearance of broad peaks at around 900–1100 cm^–1^ indicates the coordination of bipyridyl ligands to the Co–Fe structures (Figure 5a), while the small band in the cyanide region (∼2100 cm^–1^) is ascribed to structural changes in the cyanide environment due to the coordination of bipyridyl groups to cobalt sites (Figure 5b). Almost a twofold increase in the atomic percent of the pyridyl-N ( N- py) peak at 398.95 eV in the N 1s signal of the postcatalytic XPS spectra (Figure 4c,f) also suggests the presence of an additional pyridyl group in the structure that could only arise from bipyridyl poisoning. A small Ru 3d core-level XPS peak is also observed at 280.5 eV in the postcatalytic XPS spectra, which confirms the degradation of [Ru(bpy)_3_]^2+^ complex (Figure S8a,c). The saturation in the activity after 1 h of light exposure (Figure S9), therefore, could be attributed to the decomposition of the ruthenium complex, followed by the inhibition of the catalytic cobalt sites.

Overall, postcatalytic studies show that cyanide-based Co–Fe systems are robust under photocatalytic conditions and their water oxidation activities are greatly influenced by the incorporation of pyridyl groups into the coordination environment of the catalytic sites.

### Evaluation of TOF

We performed comprehensive characterization and electrochemical studies to elucidate the effect of pyridyl groups on morphological and electronic properties of the Co–Fe compounds. For this purpose, turnover frequencies (TOFs) of compounds were estimated with two different methods, which differ mainly in the estimation of the active cobalt sites (see eqs S1–S6 for details of calculations): lower-bound TOF (TOF_lb_) and upper-bound TOF (TOF_ub_). In the first approach, all of the cobalt sites, even the cobalt ions in the bulk of the PB network structure, are assumed to be active to estimate the number of moles of active sites. The TOF_lb_ values attained in the first 15 min of photocatalysis for **[Cophen–Fe]**, **[Cobpy–Fe]**_,_ and **[Co–Fe]** were estimated as 4.35 × 10^–4^, 3.91 × 10^–4^, and 1.73 × 10^–4^ s^–1^, respectively, which are comparable to the previously reported ones for PBA-based catalysts.^6,10^ This method reveals that the activity of cobalt sites increases at least two times when pyridyl groups are introduced to its coordination sphere.

**Figure 6:**
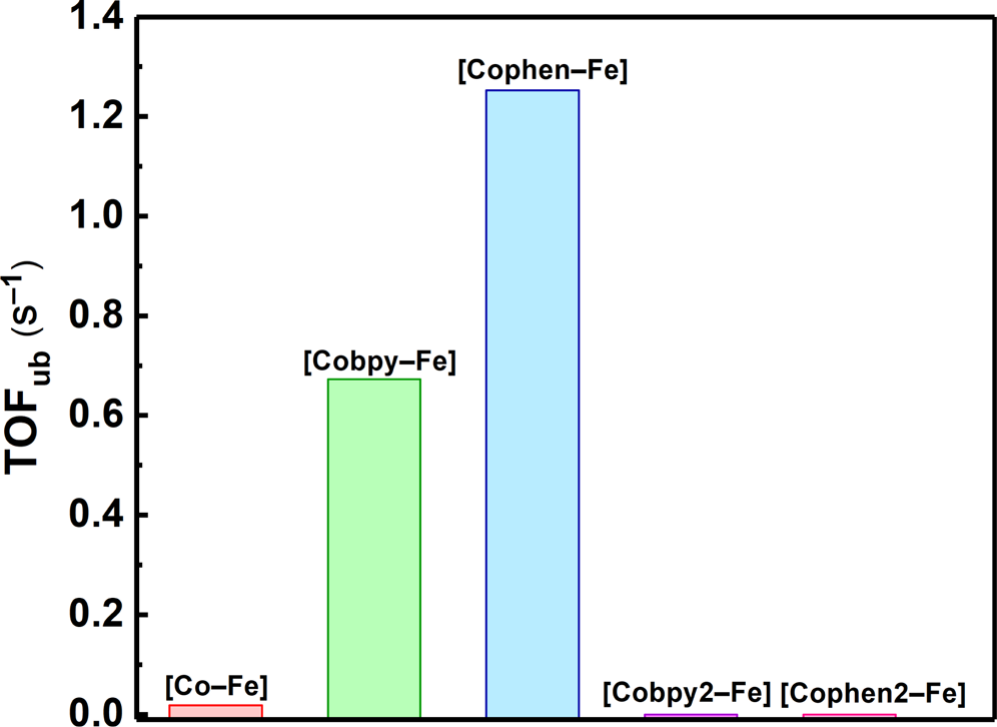
TOF_ub_ obtained by the compounds in the first 15 min of photocatalysis.

In the second method, cyclic voltammetry experiments were performed at different scan rates (50–300 mV·s^–1^) to extract the surface concentration (Γ) of active cobalt sites from the electrochemical linear dependence between peak current (*I*) of the Co^3+^/Co^2+^ reduction wave and the scan rate (ν).^3^ Surface concentration of 10.01, 0.53, and 0.32 nmol·cm^–2^ are found for **[Co–Fe]**, **[Cobpy–Fe]**, and **[Cophen–Fe]**, respectively (Figure S10). The significant decrease in the surface concentration implies that the bulky bidentate pyridyl groups block the surface-active sites. The unfavorable blocking of the cobalt sites with bidentate pyridyl groups is, however, not reflected on the catalytic activities of **[Cobpy–Fe]** and **[Cophen–Fe]**, indicating that electronic parameters play a more prominent role than the morphological properties in the photocatalytic process. The TOF_ub_ values, which are based on surface-active cobalt sites, reveal the remarkable electronic effect of the bidentate pyridyl groups. The TOF_ub_ of **[Cobpy–Fe]** (0.7 s^–1^) and **[Cophen–Fe]** (1.3 s^–1^) are markedly superior to that of **[Co–Fe]** (1.8 × 10^–2^ s^–1^) by a factor of approximately 37 and 70, respectively (Figure 6). The TOF_ub_ depicts a realistic value to the actual TOF because only the surface sites are active to catalysis in heterogeneous catalysis, the bulk catalyst is inactive. Furthermore, it gives insights into the catalytic activity of the individual surface cobalt atoms. These TOF values are compared with those achieved by other methods of enhancing the photocatalytic activity of the cobalt catalyst (Table 1).

**Table 1:** Comparison of Water Oxidation TOF of Various Cobalt Catalyst and Prussian Blue Analogue-Based Compounds

catalyst	lower-bound TOF × 10^–4^ (s^–1^)	upper-bound TOF (s^–1^)	ref
Co–Fe PBA	1.7	0.018	this work
Cobpy–Fe	3.9	0.7	this work
Cophen–Fe	4.4	1.3	this work
nano-SiO_2_/Co_3_O_4_	3.3		(37)
nano-Al_2_O_3_/Co_3_O_4_	4.61		(37)
cobalt oxide nanocubane		0.023	(38)
cobalt–phosphate (Co–Pi)		0.053, 0.105	(39)
Co–Fe PBA	3	0.0023	(6)
Co–Co PBA	5.3	0.0032	(6)
Mn–Fe PBA	2.2		(6)
CoFe–TPyP	3.2		(40)
LDH-PB	2.1		(10)

The significant enhancement in TOF parameters of **[Cobpy–Fe]** and **[Cophen–Fe]** reveals that controlled tuning of the coordination sphere of the cobalt site increases the rate of the water oxidation process. By replacing two of the weak π-accepting −NC ligands around the cobalt sites in **[Co–Fe]** with one equivalent of stronger π-accepting phen and bpy ligands, the strong withdrawing ability increases the electrophilicity and susceptibility of the high-valent catalytic Co(IV)-oxo species to the nucleophilic attack of water for O_2_ formation. As shown in several studies on water oxidation mechanistic analysis,^41,42^ the nucleophilic attack of water on the high-valent catalytic Co(IV)-oxo species is the rate-determining step for water oxidation. Furthermore, a close competition between the activity of **[Cophen–Fe]** and **[Cobpy–Fe]** is observed although phen as a more π-accepting ligand than bpy,^43^ should increase the electrophilicity of the high-valent Co(IV)-oxo species. A possible explanation could be because bpy is substantially more σ-donating than phen.^44^ This σ-donating ability of bpy could increase the electron density on the cobalt site of **[Cobpy–Fe]**, which favors the formation of a stable electron-deficient high-valent Co(IV)-oxo species.^45^

### Electronic Structure Calculations

**Figure 7:**
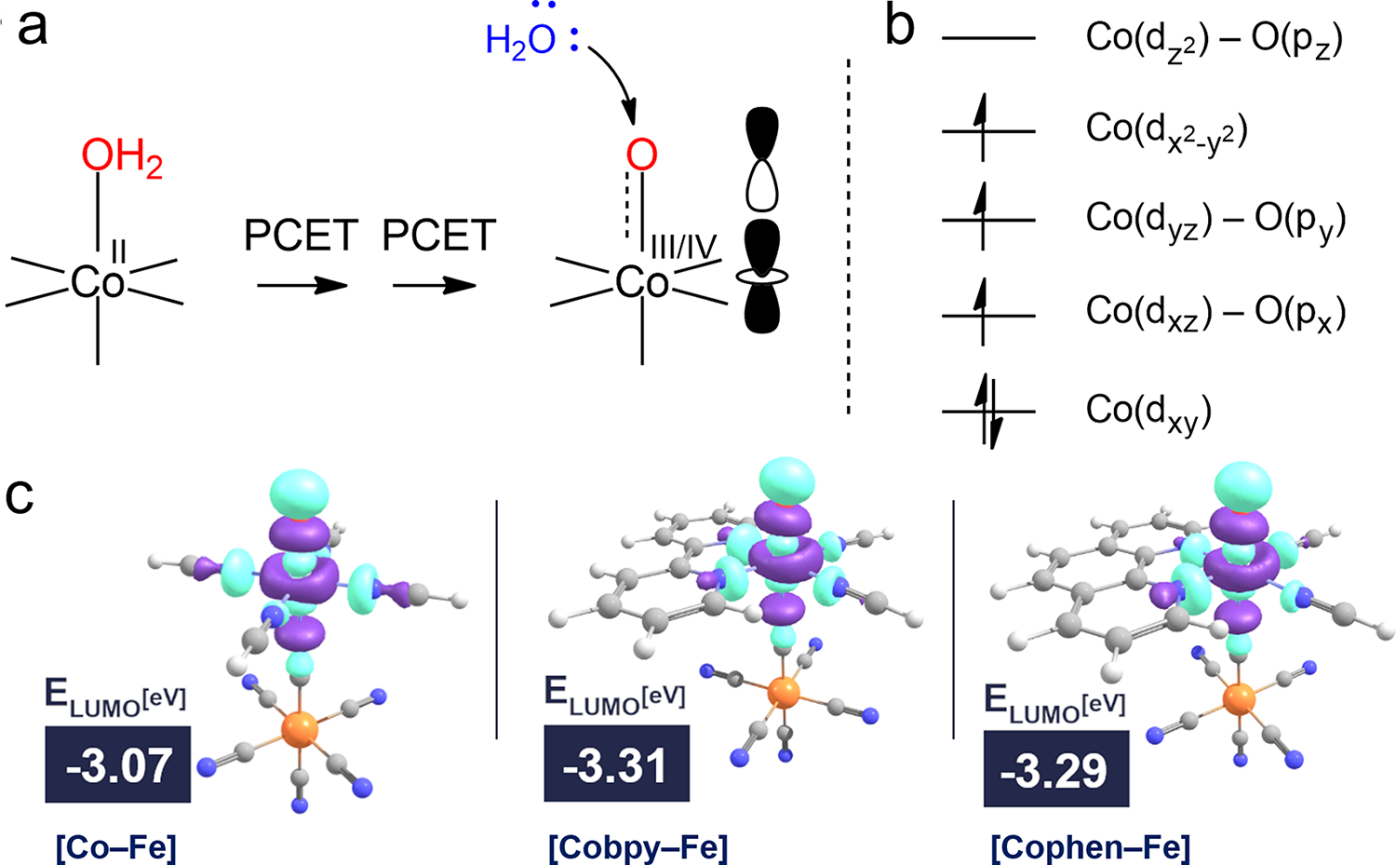
(a) PBAs proceeding through PCET steps to afford catalytically active Co(IV)-oxo/Co(III)-oxyl moiety, with incoming water attacking the Co–oxygen moiety. (b) Electronic structure of Co–oxygen moiety. (c) Lowest unoccupied molecular orbital (LUMO) energies and orbital distributions for **[Co–Fe]**, **[Cophen–Fe]**, and **[Cobpy–Fe]**.

The effect of bidentate pyridyl ligands on the Co center was further investigated with electronic structure calculations. The suggested mechanism in the literature along with our previous works^46−49^ suggests that the oxidation of water would require a Co^IV^-oxo/Co^III^-oxyl moiety. PB-based water oxidation catalytic process presumably proceeds through proton-coupled electron transfer (PCET) steps to afford the Co(IV)-oxo/Co(III)-oxyl structure, i.e., Co^II^(OH_2_)→Co^III^(OH)→Co^IV^(O)/Co^III^(O). Incoming water then attacks the Co(IV)-oxo/Co(III)-oxyl moiety to yield the O–O bond formation (Figure 7a). Therefore, the structural and electronic features of the Co^IV^-oxo/Co^III^-oxyl are the focus of our quantum mechanical calculations.

Electronic structures of the Co^IV^–oxo/Co^III^–oxyl moiety bears a local quartet Co–oxygen center for **[Co–Fe]**, **[Cophen–Fe]**, and **[Cobpy–Fe]** (Figure 7b). Cobalt d_(*x*^2^–*y*^2^)_ orbital forms bonding and antibonding interactions with the ligand orbitals, σ[Co(d_(*x*^2^–*y*^2^)_) + ligand] and σ[Co(d_(*x*^2^–*y*^2^)_) – ligand]. d_(*z*^2^)_ analogues of the bonding/antibonding pair is then σ[Co(d_*z*^2^_) + O(p*_z_*)] and σ[Co(d_*z*^2^_) – O(p*_z_*)] molecular orbitals (MOs). The remaining d-orbitals are nonbonding, but oxygen p-orbitals form π-bonding interactions with the metal center.^50^ Consequently, the *xz* and *yz* components of the d-orbitals also form bonding and antibonding orbitals. An approximate local occupation pattern can be represented as d*_xz_*(↑↓)·σ[Co(d_(*x*^2^–*y*^2^)_) – ligand](↑)·π[Co(d*_xz_*) – O(p*_x_*)](↑)·π[Co(d*_yz_*) – O(p_y_)](↑)·σ[Co(d_*z*^2^_) – O(p*_z_*)]() (Figure 7b). The local quartet assignment of the Co center is also verified with spin density analysis. It is important to note that no restrictions are imposed on the distribution of electrons in our quantum chemical calculations.

The critical O–O-bond-forming process can be readily understood by analyzing the electronic structure. Lone pairs of oxygen in water seek available orbitals for electron transfer, for which the LUMO is the best candidate. Following the MO description above, Co–oxygen center bears three singly occupied orbitals (Figure 7b and Table S3), and LUMO is composed of Co(d_*z*^2^_) and O(p*_z_*) orbitals. One of the singly occupied orbitals, σ[Co(d_(*x*^2^–*y*^2^)_) – ligand], resides mainly on the cobalt center. The remaining singly occupied orbitals are centered on the Co–O bonding axis and orthogonal to each other.

In this quartet electronic structure of the Co–oxygen center, the reported reactivities and energies of the LUMO should be correlated since oxygen lone pairs of water are proposed to fill in the LUMO on Co–oxygen center. In particular, lower LUMO energies should yield higher catalytic activities.^16,42,46,51^ Furthermore, **[Cobpy2–Fe]** and **[Cophen2–Fe]** compounds suffer from no activity as there is no bound Co–oxygen structure in these complexes and, therefore, no available orbitals for the electron transfer. Note, however, that catalytic activity stems from numerous complex, molecular, and applicational features and our MO analyses here attempt to uncover the electronic structure causes of the activity.

Orbital distributions and energies of LUMO for the complexes are given in Figure 7c. In line with our MO arguments, LUMO is obtained at lower energies for **[Cobpy–Fe]** and **[Cophen–Fe]** (−3.31 and −3.29 eV relatively) compounds compared to **[Co–Fe]** (−3.07 eV). The electron affinity of the Co–oxygen center is hence increased when the aromatic ligands, bpy or phen, are coordinated to the Co center. Consequently, the attack of water becomes more facile in the presence of aromatic ligands.

Note that our quantum chemical calculations did not use the complete molecular structure of PB surface, but the electronic structure calculations and our experimental results were in excellent agreement and, therefore, the truncated model used herein is beneficial.

## Conclusions

In summary, we showed that the intrinsic activity of the Co–Fe PBA catalyst could be tuned by the coordination of bidentate pyridyl groups to the catalytic cobalt sites. Cobalt-mono(bipyridyl) precursors are reacted with hexacyanoferrate complex to prepare **[Cobpy–Fe]** and **[Cophen–Fe]**, which possess cobalt sites coordinated pyridyl and −NC groups as well as water molecules. In **[Cobpy–Fe]** and **[Cophen–Fe]**, structures with lower-dimensionality and less crystalline nature are observed compared to regular **[Co–Fe]**. These compounds also exhibit a higher number of Co^3+^ sites compared to **[Co–Fe]**, which is reflected in their photocatalytic water oxidation activities. A concise summary of the characterization of all samples is shown in Table S4. Due to the well-tuned electronic effect caused by the electron-withdrawing bidentate pyridyl groups, the photocatalytic activities of **[Cobpy–Fe]** and **[Cophen–Fe]** outperform **[Co–Fe]**. The electronic effect generated by bidentate pyridyl ligand coordination foster water oxidation by (i) increasing the electrophilicity of the Co(IV)-oxo species to the nucleophilic attack of water through their strong π-accepting ability and (ii) sufficiently stabilizing the highly valent Co(IV) state by strong sigma donation and bidentate coordination.

Electronic structure calculations supported experimental observations by confirming that the coordination of bidentate pyridyl groups to the catalytic cobalt sites can sufficiently lower the LUMO energy barrier required for the crucial O–O bond formation in water oxidation kinetics. In addition to this enhanced activity, another interesting finding in this work is that free coordination or aqua coordination on the catalytic cobalt sites in Co–Fe PBA is essential for water oxidation. Molecular **[Cobpy2–Fe]** and **[Cophen2–Fe]** complexes designed by reacting cobalt–bis(bipyridyl) with hexacyanoferrate precursor show no oxygen evolution since the coordination sphere of cobalt sites are entirely decorated with bipyridyl and cyanide groups. This explored strategy provides an understanding of the coordination environment of catalytic sites in PBA and opens a new pathway toward optimizing the intrinsic activity of PBA-based catalysts. Our study on tuning the catalytic activity with other bidentate ligands is currently in progress.

## Supporting Information Available

UV–vis and FTIR spectra of cobalt precursors, full FTIR spectra of CoFe compounds, SEM images, TGA curves, diffraction patterns and structures, pristine, and postcatalytic XPS spectra of C 1s and O 1s signal, second cycle photocatalytic OER, cyclic voltammograms, tables showing EDX and CHN elemental analysis results, computational details and molecular orbital diagrams, the summary table of characterizations of CoFe compounds, and TOF calculations (PDF)
