# Supplementary material for: Light-Driven Water Oxidation with Ligand-Engineered Prussian Blue Analogues
Source: Inorg Chem. 2022 Feb 24;61(9):3931–41. doi: 10.1021/acs.inorgchem.1c03531 (PMC8905577; doi:10.1021/acs.inorgchem.1c03531)
Supplement: Supplementary file 1 — ic1c03531_si_001.pdf [file ic1c03531_si_001.pdf]

Supporting Information

Light-driven Water Oxidation with Ligand-Engineered Prussian Blue Analogues

Aliyu A. Ahmad,[†] *T. Gamze Ulusoy Ghobadi,*[‡] *Muhammed Buyuktemiz,*[§] *Ekmel Ozbay,*[‡,#,¶] *Yavuz Dede*[*,§] *and Ferdi Karadas*[*,†,‖]

[†]Department of Chemistry, Faculty of Science, Bilkent University 06800 Ankara, Turkey

[‡]NANOTAM—Nanotechnology Research Center, Bilkent University, 06800 Ankara, Turkey

[§]Department of Chemistry, Faculty of Science, Gazi University Teknikokullar, 06500 Ankara, Turkey

[#]Department of Electrical and Electronics Engineering, Bilkent University, 06800 Ankara, Turkey

[¶]Department of Physics, Faculty of Science, Bilkent University 06800 Ankara, Turkey

[‖]UNAM—National Nanotechnology Research Center, Bilkent University, 06800 Ankara, Turkey

*Email: karadas@fen.bilkent.edu.tr (F.K.)

*Email: dede@gazi.edu.tr (Y.D.)

Table of Contents **Page**

Figures S3

Tables S13

Calculations S18

References S19

Figures

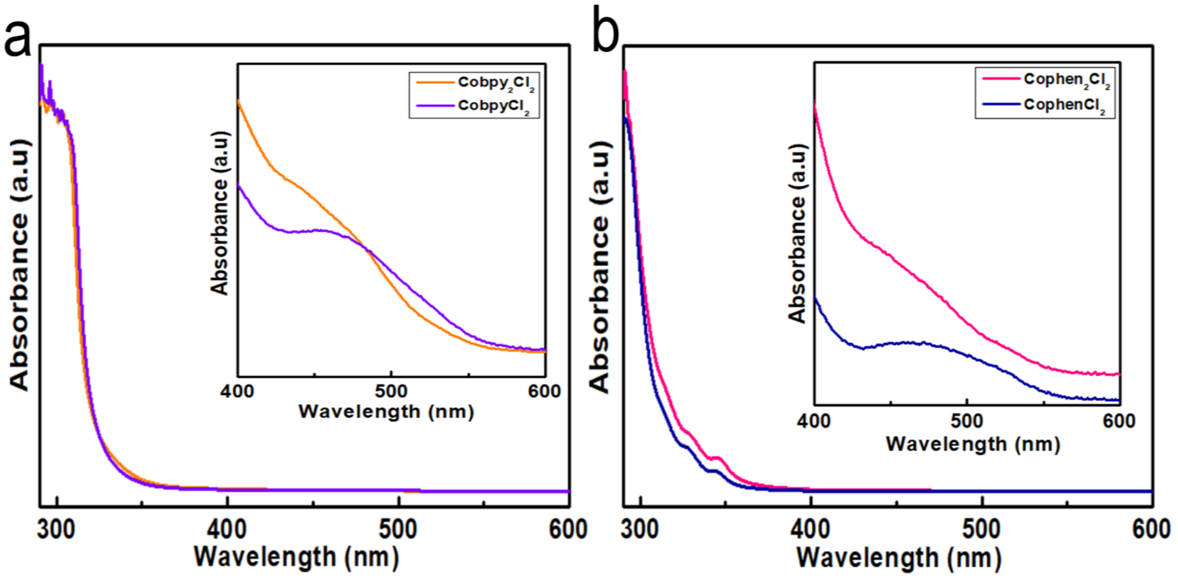

Figure S1. UV-Vis absorption spectra of an aqueous solution of (a) $CobpyCl_2$, $Cobpy_2Cl_2$ and (b) $CophenCl_2$, $Cobpy_2Cl_2$. Inset: absorption bands in the visible region of the spectra (400 - 600 nm).

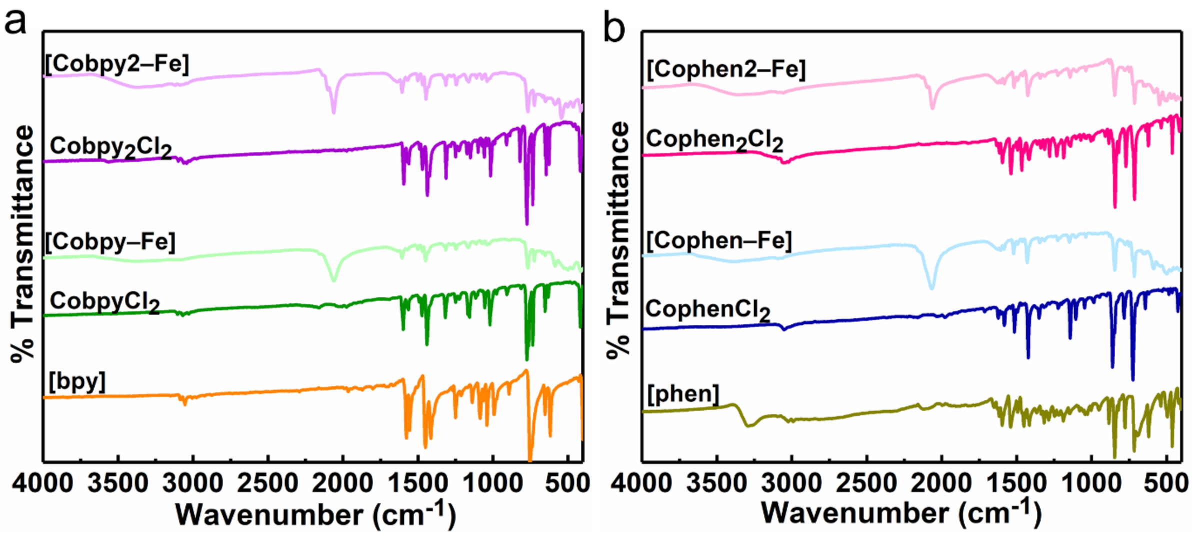

Figure S2. (a) FTIR spectra of Cobalt bipyridine precursors and their corresponding CoFe compounds. (b) FTIR spectra of cobalt phenanthroline precursors and their related CoFe compounds.

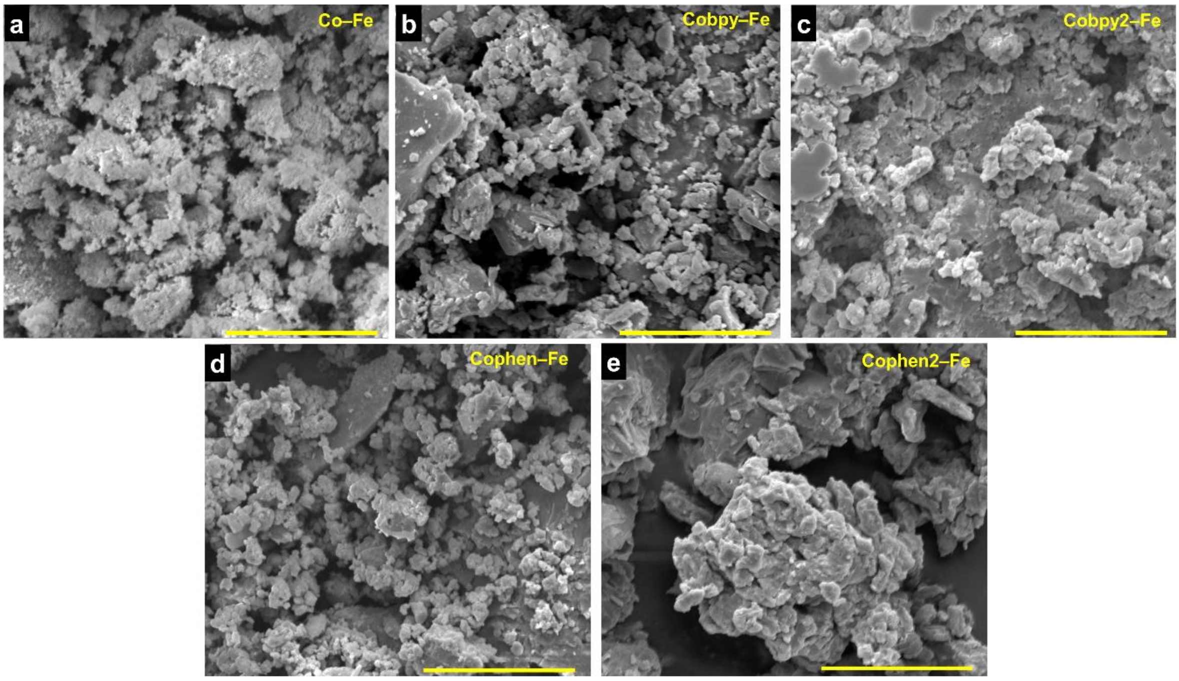

Figure S3. SEM micrographs of (a) [**Co–Fe**], (b) [**Cobpy–Fe**], (c) [**Cobpy2–Fe**], (d) [**Cophen–Fe**], and (e) [**Cophen2–Fe**]**.** Scale bar: 10μm.

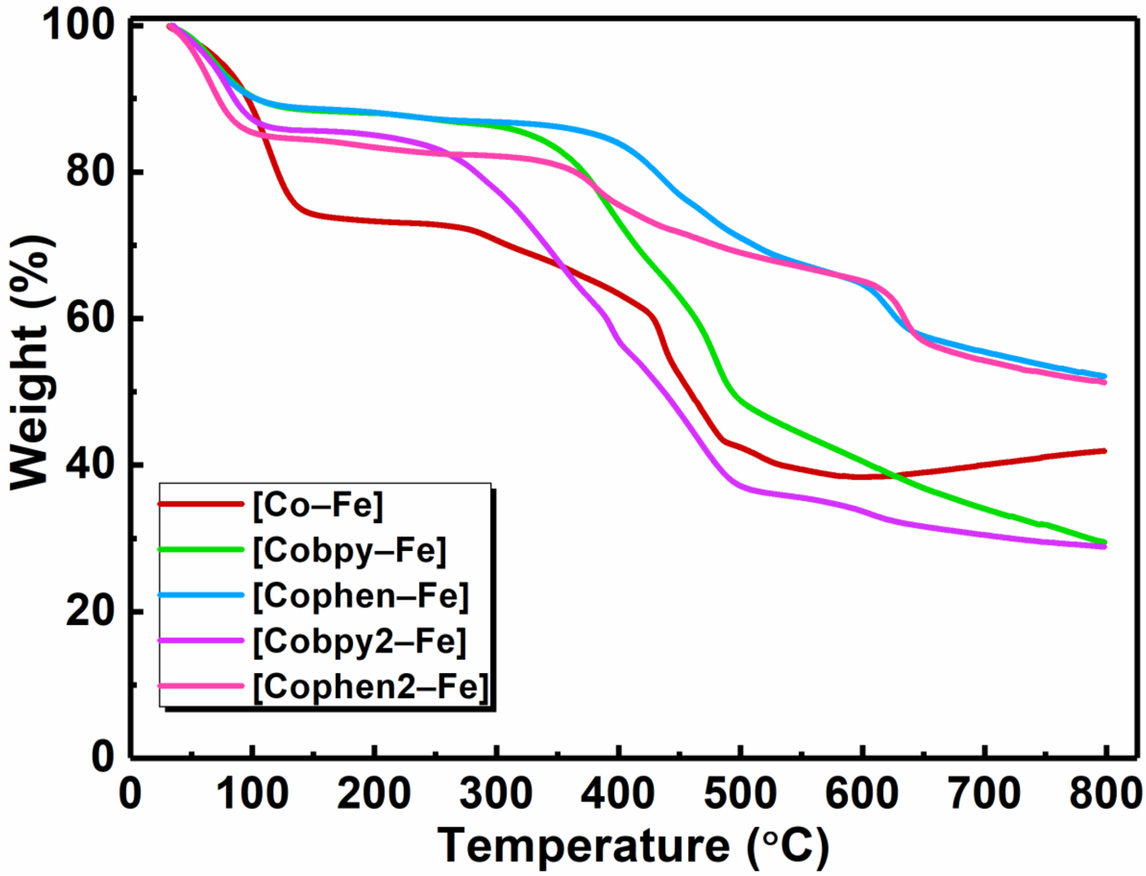

Figure S4. Thermogravimetric curves of CoFe compounds. The thermal analysis was performed under N_2 atmosphere in the temperature range 30 °C to 800 °C at a heating rate of 5 °C/min. The decomposition event at 30 °C to 175 °C is attributed to the loss of coordinated and non-coordinated water molecules, while the rest of the decomposition events at temperatures > 250 °C is due to the decomposition and transformation of the cyanide network to oxides.[1]

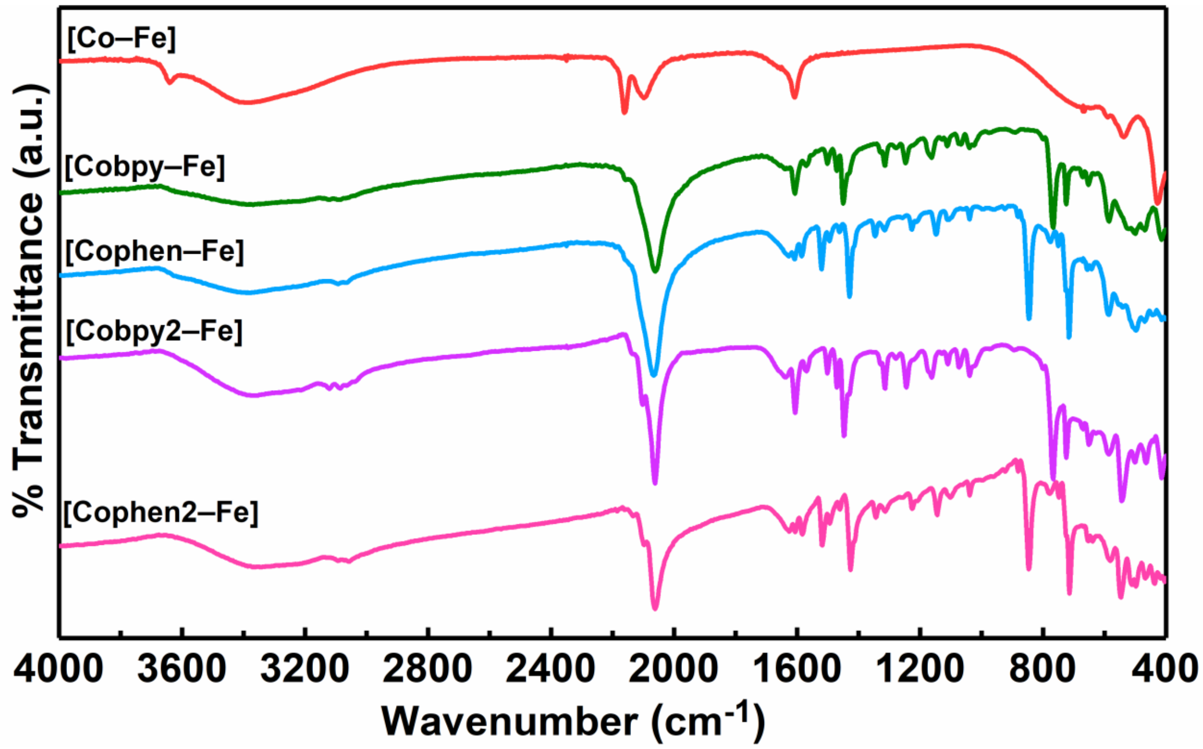

Figure S5. Full FTIR spectra of CoFe compounds at room temperature. The comparison of the bands in the fingerprint region of [**Co-Fe**] and Co(pyridyl)-Fe compounds reveals the bidentate pyridyl ligands are well coordinated to the cobalt sites

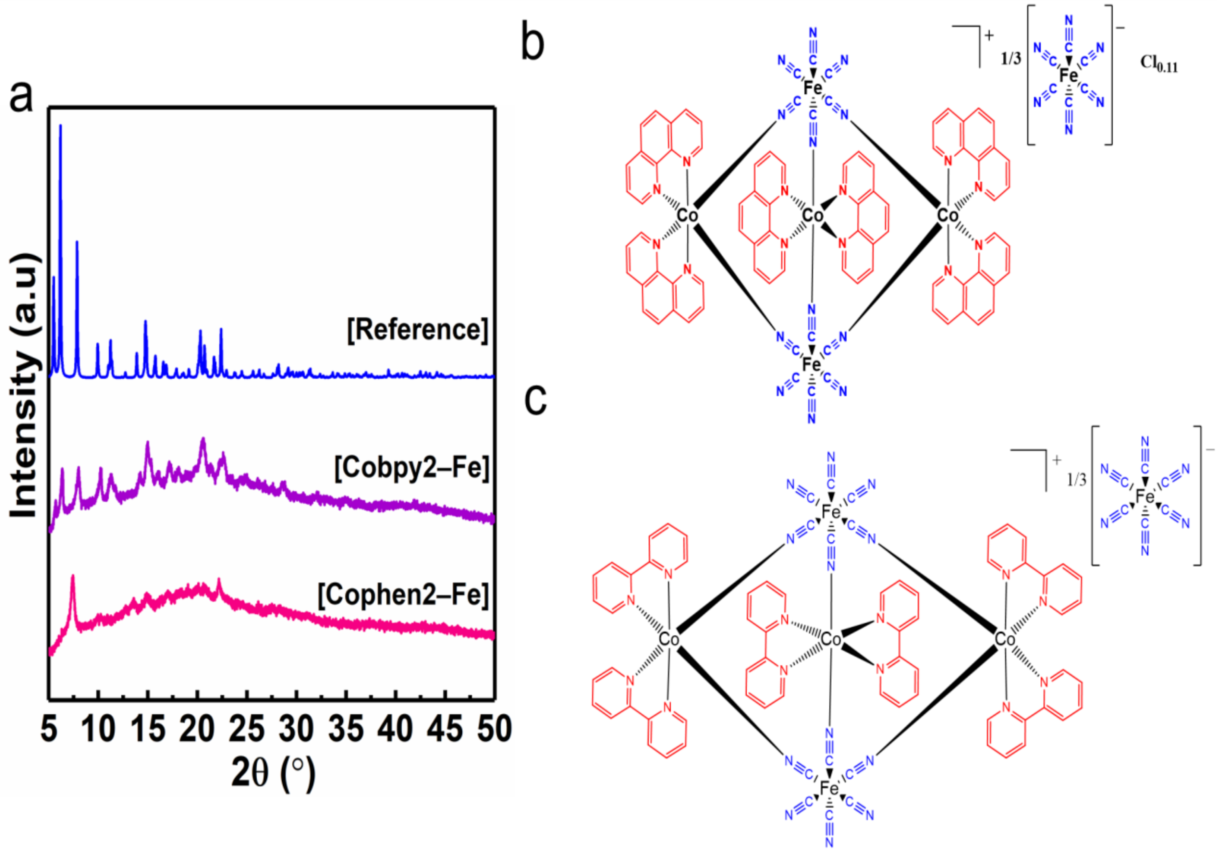

Figure S6. (a) PXRD patterns of [**Cobpy2–Fe**] and [**Cophen2–Fe**] matched with a reference compound. The reference is a previously synthesized molecular trigonal bipyramidal Co_3Fe_2 cluster {[Co(bpy)$_2$]$_3$[Fe(C.N.)$_6$]$_2$}[Fe(C.N.)$_6$]$_{1/3}$.[2] Although, [**Cophen2–Fe**] and [**Cobpy2–Fe**] are both trigonal bipyramidal compounds[3], the position of the diffraction peaks are slightly altered and are broader in [**Cophen2–Fe**] compared to [**Cobpy2–Fe**], which is probably due to the difference in the size of phen and bpy ligands and the type of the counterion. Trigonal bipyramidal molecular structures of (b) [**Cophen2–Fe**] **and** (c) [**Cobpy2–Fe**].

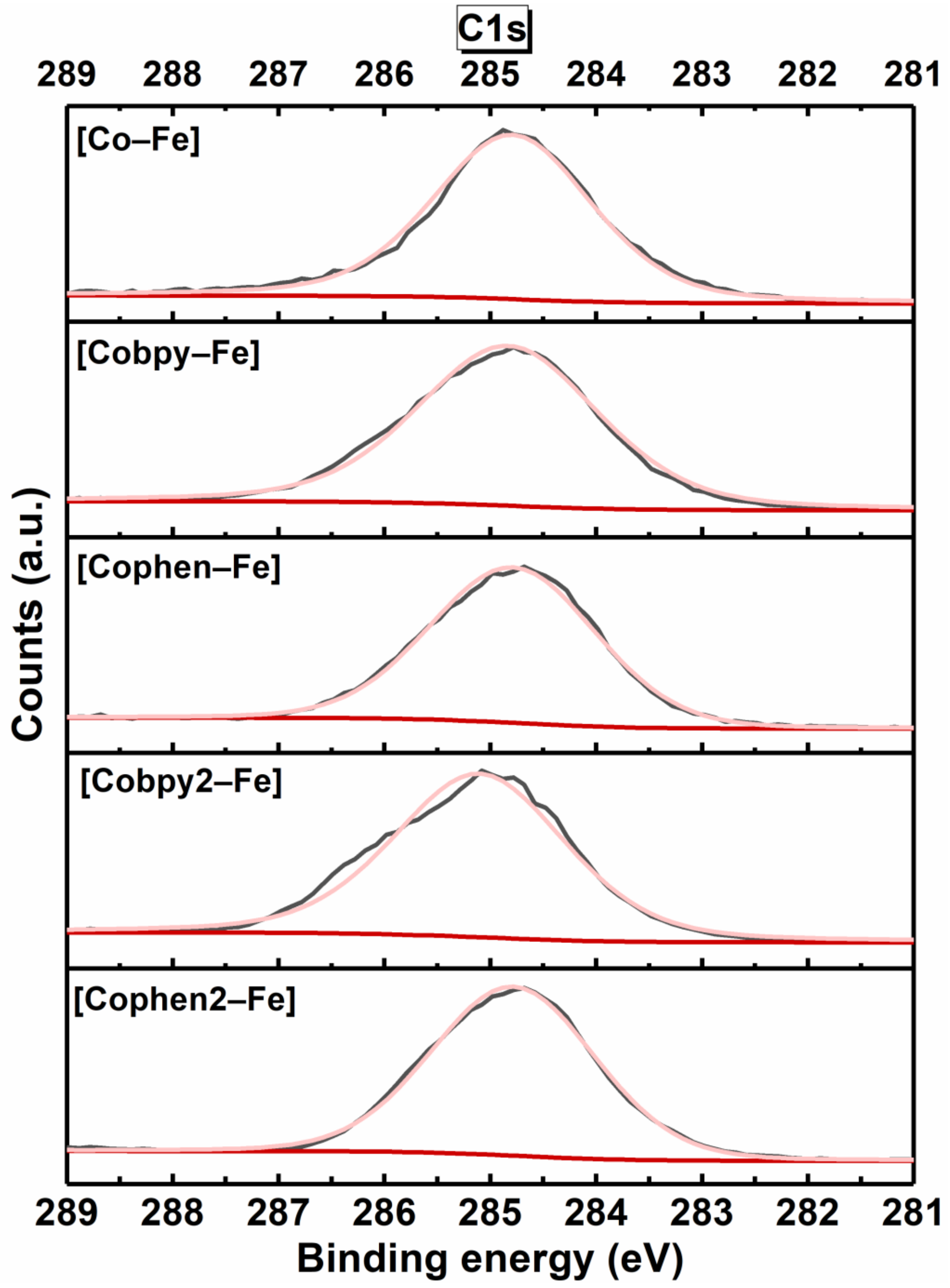

Figure S7. High resolution XPS spectra of C1s signal for the CoFe compounds.

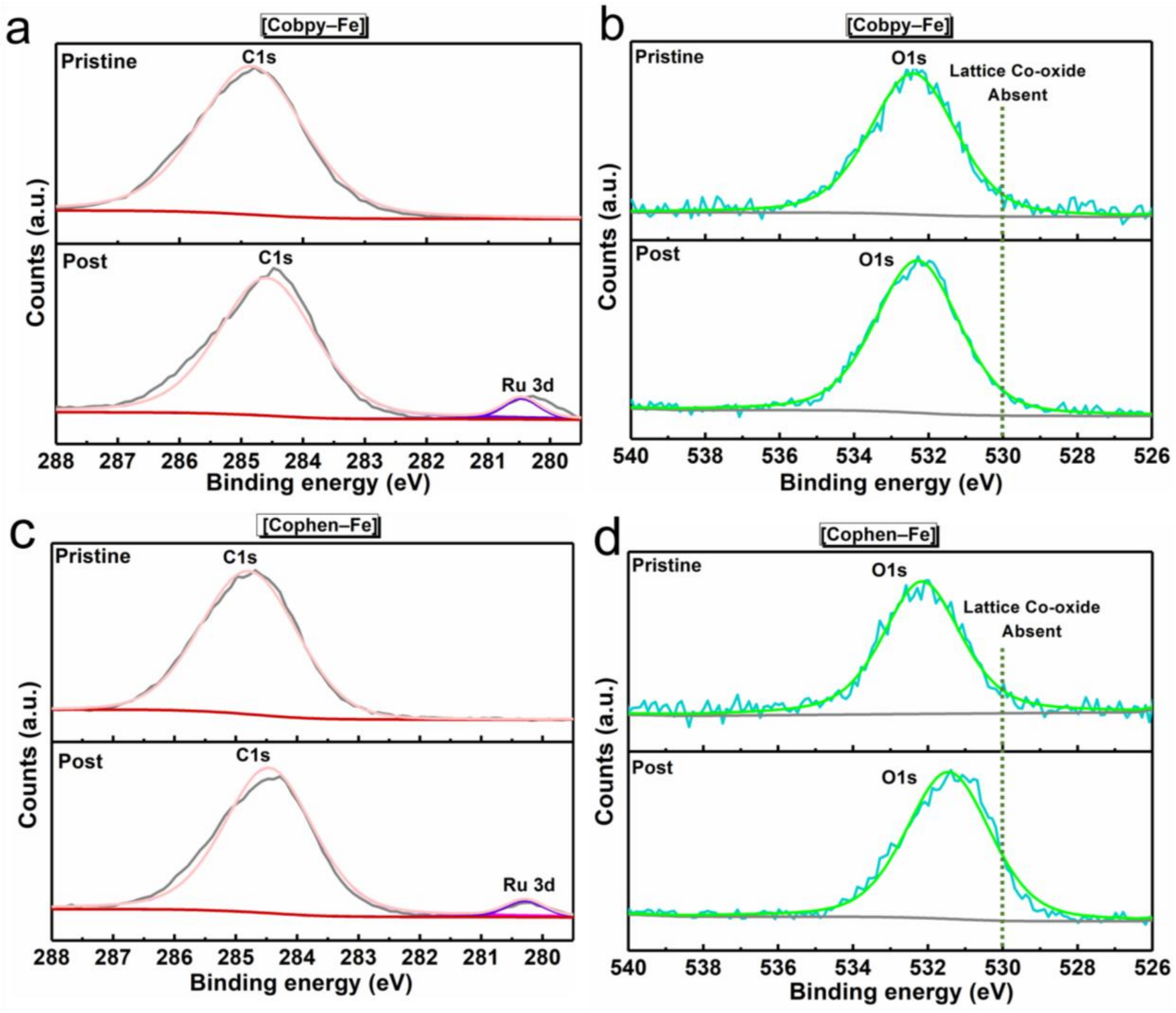

Figure S8. (a) C1s and (b) O1s Pristine and Post-catalytic high resolution XPS spectra for [**Cobpy–Fe**]. (c) C1s and (d) O1s pristine and post-catalytic high resolution XPS spectra for [**Cophen–Fe**].

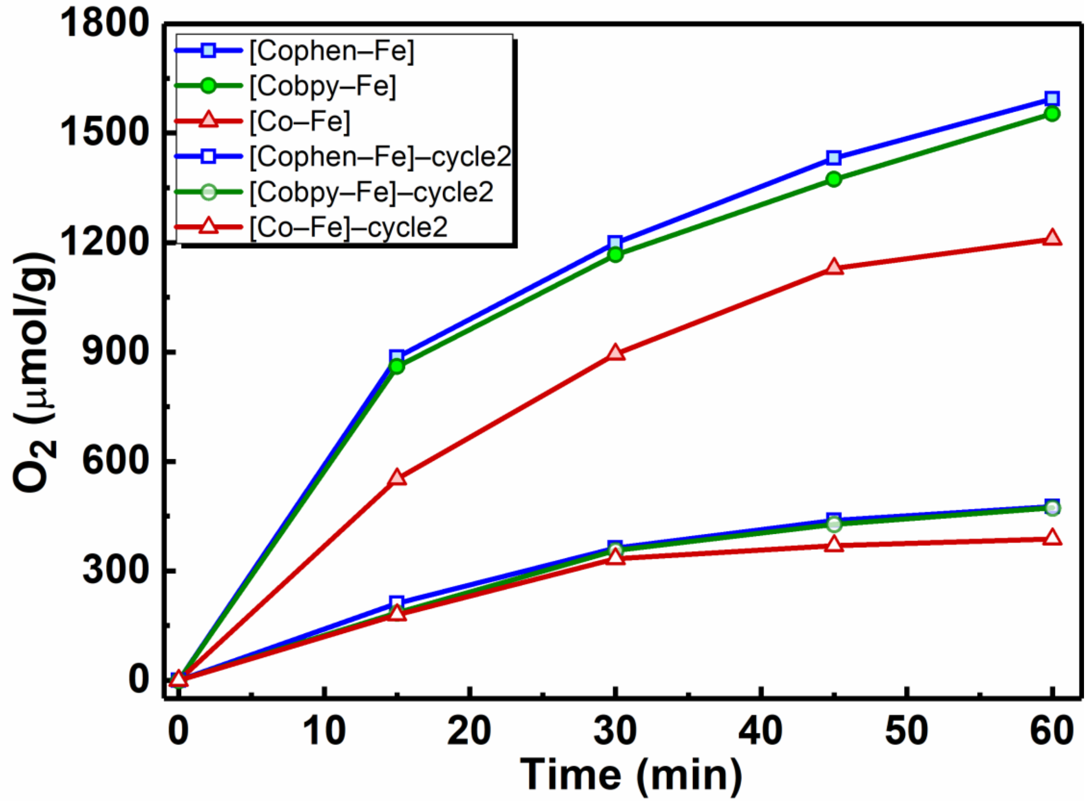

Figure S9. Photocatalytic oxygen evolution reaction activities of [**Co–Fe**], [**Cobpy–Fe**] and [**Cophen–Fe**] for the 1st and 2nd cycles. Experimental conditions: an aqueous phosphate buffer solution (pH 7) containing 10 mg catalyst, 1 mM $[Ru(bpy)_3]^{2+}$ photosensitizer, and 5 mM $Na_2S_2O_8$ as the sacrificial electron scavenger. Fresh 1 mM $[Ru(bpy)_3]^{2+}$ and 5 mM $Na_2S_2O_8$ were added into the solution at the beginning of cycle 2.

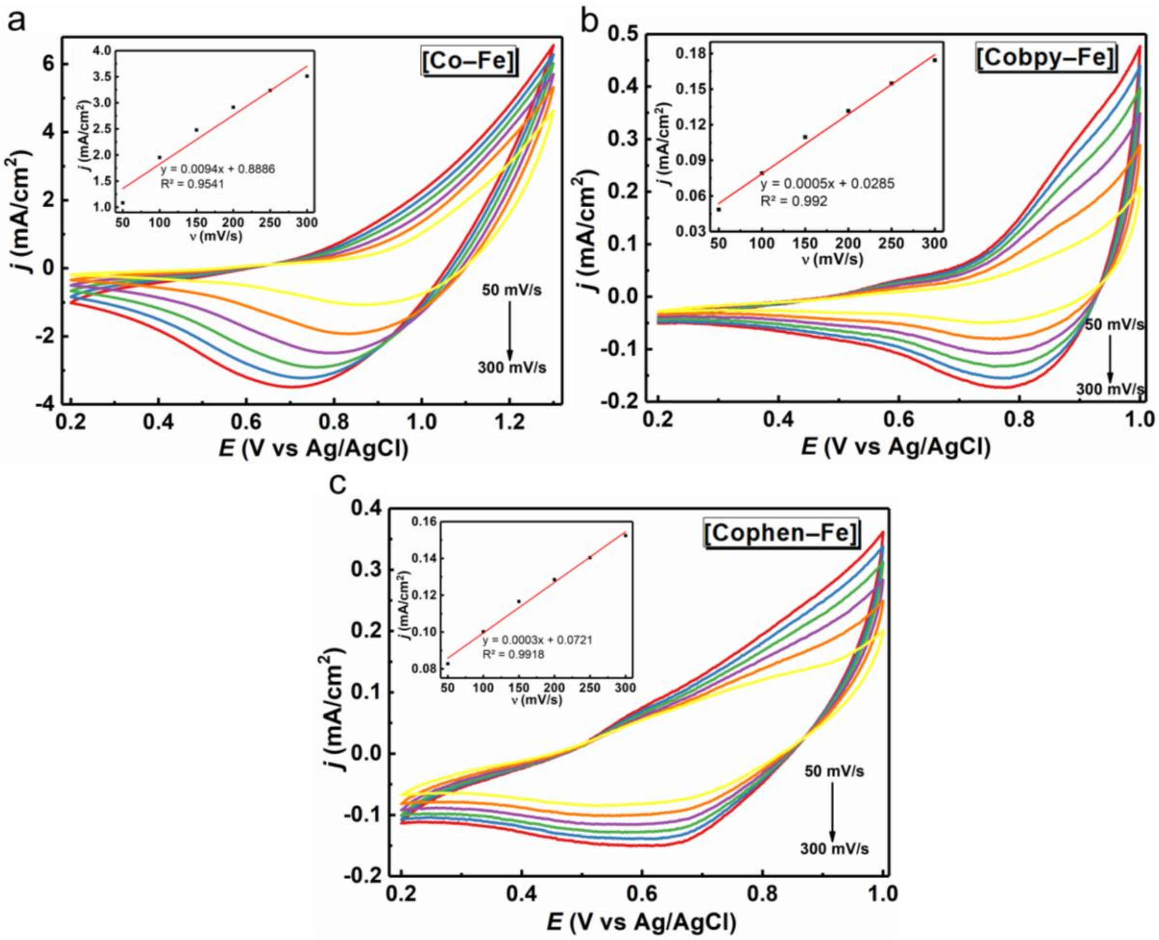

Figure S10. Cyclic voltammogram of (a) [**Co–Fe**], (b) [**Cobpy–Fe**] and (c) [**Cophen–Fe**] recorded at different scan rate ranging from 50 - 300 mV/s. Inset: linear plot of the peak current (I) of Co^{3+}/Co^{2+} reduction wave versus scan rate (v). The CV experiments were performed in a potassium phosphate buffer (KPi) solution at pH 7 containing 1 M KNO_3 as the supporting electrolyte.

Tables

Table S1. EDX elemental analysis of CoFe compounds and their obtained chemical formulas

Compound	EDX Atomic %							TGA	Chemical Formula
	Co	Fe	C	N	O	K	Cl	% Water molecule	
[Co–Fe]	2.91	2.01	58.5	28.28	7.43	0.1	–	26.58	$K_{0.1}Co_{2.9}[Fe(CN)_6]_2 \cdot 12H_2O$
[Cobpy–Fe]	2.97	2.04	68.62	23.7	1.94	0.31	–	11.87	$K_{0.1}[Co(bpy)]_{2.9}[Fe(CN)_6]_2 \cdot 7.5H_2O$
[Cophen–Fe]	3.63	2.6	70.21	21.44	1.06	0.53	–	11.34	$K_{0.2}[Co(phen)]_{2.8}[Fe(CN)_6]_2 \cdot 7.5H_2O$
[Cobpy2–Fe]	1.79	1.47	68.13	27.61	0.55	–	–	14.32	$\{[Co(bpy)_2]_3[Fe(CN)_6]_2\}[Fe(CN)_6]_{1/3} \cdot 14.5H_2O$
[Cophen2–Fe]	2.11	1.65	75.79	18.09	1.13	–	0.62	15.48	$\{[Co(phen)_2]_3[Fe(CN)_6]_2\}[Fe(CN)_6]_{1/3}Cl_{0.11} \cdot 17.5H_2O$

Table S2. CHN elemental analysis of the studied CoFe compounds. The results obtained in CHN analysis agree with the elemental ratio determined from EDX analysis and the percentage of water molecules obtained from thermal analysis.

Compound	Chemical Formula	Calculated / Found(CHN)		
		%C	%N	%H
[Co–Fe]	$K_{0.1}Co_{2.9}[Fe(CN)_6]_2 \cdot 12H_2O$	17.69 / 17.19	20.61 / 19.52	2.94 / 2.84
[Cobpy–Fe]	$K_{0.1}[Co(bpy)]_{2.9}[Fe(CN)_6]_2 \cdot 7.5H_2O$	41.45 / 41.04	20.99 / 20.16	3.21 / 2.85
[Cophen–Fe]	$K_{0.2}[Co(phen)]_{2.8}[Fe(CN)_6]_2 \cdot 7.5H_2O$	44.25 / 43.17	19.92 / 18.98	3.02 /2.80
[Cobpy2–Fe]	$\{[Co(bpy)_2]_3[Fe(CN)_6]_2\}[Fe(CN)_6]_{1/3} \cdot 14.5H_2O$	47.49 / 46.08	19.47 / 18.53	4.06 / 3.72
[Cophen2–Fe]	$\{[Co(phen)_2]_3[Fe(CN)_6]_2\}[Fe(CN)_6]_{1/3}Cl_{0.11} \cdot 17.5H_2O$	49.81 / 47.58	17.57 / 17.28	4.01 / 3.23

Table S3. Orbital plots for the singly occupied molecular orbitals of [**Co–Fe**], [**Cobpy–Fe**], and [**Cophen–Fe**] using UB3LYP/cc-pVTZ level of theory

MO	[Co–Fe]	[Cobpy–Fe]	[Cophen–Fe]
π[Co(d_{xz}) – O(p_x)]	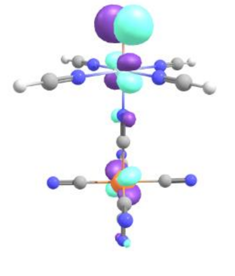	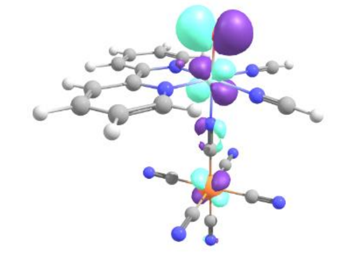	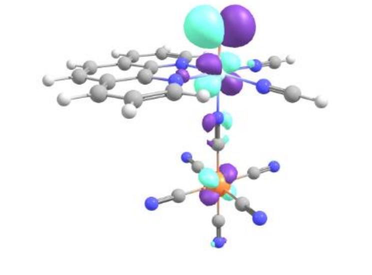
π[Co(d_{yz}) – O(p_y)]	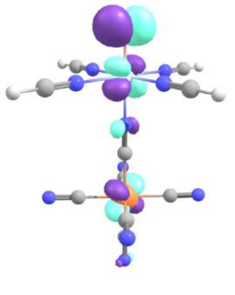	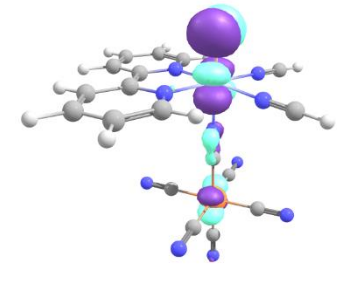	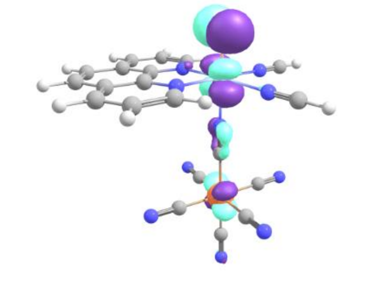
Co($d_{x^2-y^2}$)	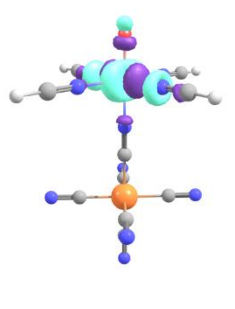	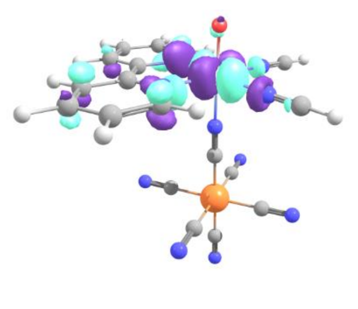	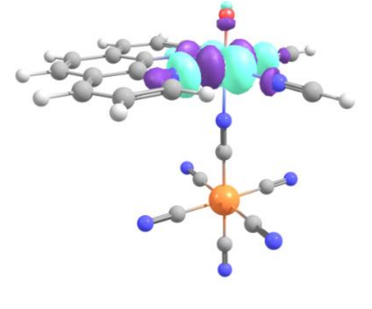

Density Functional Theory (DFT)[5–7] is used for quantum chemical calculations as implemented in the Gaussian 09 software suit.[8] Geometries were fully optimized with B3LYP[5,9] functional and effective core potential of Hay and Wadt, LANL2DZ.[10,11] No constraints were imposed on the geometry runs. Vibrational frequency calculations on the optimized geometries ensured that the geometries correspond to the minima. Dunning's correlation consistent triple zeta basis set, cc-pVTZ,[12] was used in single point runs. This level of theory was previously found to be successful in the description of electronic states and their relative energies.[13–16] Molecular orbitals were plotted with the Chemcraft Program[17] using a contour value of 0.03.

Occupancies and the energies of the orbitals were reported within the framework of Kohn-Sham theory (Figure 7c of main text). Mulliken spin densities were also checked for all structures considered. Spin density and orbital analysis suggest that iron center possess a closed shell electronic structure, whereas Co has three singly occupied orbitals in its quartet state. This type of occupancy pattern was found to have ramifications in the reactivity associated with the water splitting (see main text).

Table S4. Summary table of CoFe compounds showing the XPS atomic ratio of Fe and Co ions, PXRD morphology, assignments of cyanide stretching frequencies, the surface concentration of active cobalt sites and the calculated TOFs.

**Compounds**	**XPS (Atomic ratio)**		**XRD**		**FTIR**		**Electrochemistry**	**Photocatalysis**	
	**Co^{2+}/Co^{3+}**	**Fe^{2+}/Fe^{3+}**	**Geometry**	**Lattice parameter, (Å)**	**υ(CN) (cm^{-1})**	**Assignment**	**Surface cobalt concentration ($nmol/cm^2$)**	**Lower bound TOF (s^{-1})**	**Upper bound TOF (s^{-1})**
**[Co–Fe]**	**2.3**	**0.5**	**Cubic**	**10.243**	**2162**<br>**2098**	**Fe^{3+}–CN–Co^{2+}**<br>**Fe^{2+}–CN–Co^{2+}**	**10.01**	**1.73×10^{-4}**	**0.0181**
**[Cobpy–Fe]**	**0.49**	**2.03**	**Distorted Cubic**	**10.011**	**2058 (very broad)**<br>**2156**	**Fe^{2+}–CN & Fe^{2+}–CN–Co^{3+}**<br>**Fe^{3+}–CN–Co^{2+}**	**0.53**	**3.91×10^{-4}**	**0.6725**
**[Cophen–Fe]**	**0.62**	**1.97**	**Distorted Cubic**	**10.051**	**2058 (very broad)**<br>**2156**	**Fe^{2+}–CN & Fe^{2+}–CN–Co^{3+}**<br>**Fe^{3+}–CN–Co^{2+}**	**0.32**	**4.35×10^{-4}**	**1.252**

					2063	Fe^{2+}–CN			
[Cobpy2–Fe]	0.51	0.79	Trigonal bipyrimidal	–	2098	Fe^{3+}–CN	–	0	0
					2135	Fe^{2+}–CN–Co^{3+}			
					2063	Fe^{2+}–CN			
[Cophen2–Fe]	0.5	0.66	Trigonal bipyrimidal	–	2098	Fe^{3+}–CN	–	0	0
					2135	Fe^{2+}–CN–Co^{3+}			

`**Calculation of turn over frequencies (TOFs) of O_2 evolution**

1) **Lower bound TOF (TOF_{lb}):** The TOF was calculated by assuming that **all the cobalt sites** in the compounds are active to catalysis. The method of the calculation is given below:

10 mg of catalyst was used, therefore:

$$mass\ of\ Co\ in\ catalyst = \%\ mass\ of\ Co\ in\ catalyst \times 10\ mg$$

(eq. S1)

$$moles\ of\ Co\ in\ catalyst = \frac{mass\ of\ Co\ in\ catalyst}{molecular\ weight\ of\ Co}$$

(eq. S2)

After determining the moles of cobalt present in the catalyst, the TOF and TON was calculated as follows:

$$Turn\ over\ number(TON) = \frac{moles\ of\ O_2\ evolved\ from\ photocatalysis}{moles\ of\ cobalt\ in\ catalyst}$$

(eq. S3)

$$TOF = \frac{TON}{reaction\ time}$$

(eq. S4)

2) **Upper bound TOF (TOF_{ub}):** The TOF was calculated by assuming that **only the surface cobalt sites** in the compounds are active to catalysis. The method of the calculation is give below:

First, the surface concentration (Γ) of active cobalt sites is from the electrochemical linear dependence between peak current (I) of the Co^{3+}/Co^{2+} reduction wave and the scan rate (v).[4]

Then, the surface concentration of cobalt sites in 10 mg catalyst was obtained using the relation below:

$$= \frac{surface\ concentation\ (nmol/cm^2) \times catalyst\ eletrode\ area\ (cm^2)}{catalyst\ mass\ loading\ on\ electrode\ surface\ (mg)} \times 10\ mg$$

(eq. S5)

$$Turn\ over\ number(TON) = \frac{moles\ of\ O_2\ evolved\ from\ photocatalysis}{cobalt\ surface\ concetration\ in\ 10\ mg\ catalyst\ (nmol)}$$

(eq. S6)

The TOF was calculated using the same expression as eq. S4.

REFERENCES

(1) Wang, Q.; Wang, N.; He, S.; Zhao, J.; Fang, J.; Shen, W. Simple Synthesis of Prussian Blue Analogues in Room Temperature Ionic Liquid Solution and Their Catalytic Application in Epoxidation of Styrene. *Dalt. Trans.* **2015**, *44* (28), 12878–12883. https://doi.org/10.1039/c5dt01762a.

(2) Berlinguette, C. P.; Dragulescu-Andrasi, A.; Sieber, A.; Güdel, H. U.; Achim, C.; Dunbar, K. R. A Charge-Transfer-Induced Spin Transition in a Discrete Complex: The Role of Extrinsic Factors in Stabilising Three Electronic Isomeric Forms of a Cyanide-Bridged Co/Fe Cluster. *J. Am. Chem. Soc.* **2005**, *127* (18), 6766–6779. https://doi.org/10.1021/ja043162u.

(3) Funck, K. E.; Hilfiger, M. G.; Berlinguette, C. P.; Shatruk, M.; Wernsdorfer, W.; Dunbar, K. R. Trigonal-Bipyramidal Metal Cyanide Complexes: A Versatile Platform for the Systematic Assessment of the Magnetic Properties of Prussian Blue Materials. *Inorg. Chem.* **2009**, *48* (8), 3438–3452. https://doi.org/10.1021/ic801990g.

(4) Pintado, S.; Goberna-Ferrón, S.; Escudero-Adán, E. C.; Galán-Mascarós, J. R. Fast and Persistent Electrocatalytic Water Oxidation by Co-Fe Prussian Blue Coordination Polymers. *J. Am. Chem. Soc.* **2013**, *135* (36), 13270–13273. https://doi.org/10.1021/ja406242y.

(5) Becke, A. D. Density-Functional Exchange-Energy Approximation with Correct Asymptotic Behavior. *Phys. Rev. A* **1988**, *38* (6), 3098--3100. https://doi.org/10.1103/PhysRevA.38.3098.

(6) Frost, A. A.; Musulin, B. Density-Functional Thermochemistry. III. The Role of Exact Exchange. *Hydrocarb. J. Chem. Phys.* **1953**, *21* (August 1998), 5648.

(7) Kohn, W.; Becke, A. D.; Parr, R. G. Density Functional Theory of Electronic Structure. *J. Phys. Chem.* **1996**, *100* (31), 12974–12980. https://doi.org/10.1021/jp960669l.

(8) MJEA Frisch, GW Trucks, H Bernhard Schlegel, Gustavo E Scuseria, Michael A Robb, James R Cheeseman, Giovanni Scalmani, Vincenzo Barone, Benedetta Mennucci, GAea Petersson, H Nakatsuji, M Caricato, Xiaosong Li, HP Hratchian, Artur F Izmaylov, Julien Bloino, D. J. F. Gaussian 09, Revision d. 01, Gaussian. Inc., Wallingford CT 2009.

(9) Lee, Chengteh and Yang, Weitao and Parr, R. G. Development of the Colle-Salvetti Correlation-Energy Formula into a Functional of the Electron Density. *Phys. Rev. B* **1988**, *37* (2), 785--789. https://doi.org/10.1103/PhysRevB.37.785.

(10) Thom. H. Dunning, Ir. And Hay, P. I. Gaussian Basis Sets Tor Molecular Calculations. In *Methods of Electronic Structure Theory*; Schaefer, H. F., Ed.; Springer, Boston, MA, 1977; pp 1–27. https://doi.org/https://doi.org/10.1007/978-1-4757-0887-5.

(11) Hay, P. J.; Wadt, W. R. Ab Initio Effective Core Potentials for Molecular Calculations. Potentials for K to Au Including the Outermost Core Orbitale. *J. Chem. Phys.* **1985**, *82* (1), 299–310. https://doi.org/10.1063/1.448975.

(12) Kendall, R. A.; Dunning, T. H.; Harrison, R. J. Electron Affinities of the First-Row Atoms

Revisited. Systematic Basis Sets and Wave Functions. *J. Chem. Phys.* **1992**, *96* (9), 6796–6806. https://doi.org/10.1063/1.462569.

(13) Alsaç, E. P.; Ülker, E.; Nune, S. V. K.; Dede, Y.; Karadas, F. Tuning the Electronic Properties of Prussian Blue Analogues for Efficient Water Oxidation Electrocatalysis: Experimental and Computational Studies. *Chem. - A Eur. J.* **2018**, *24* (19), 4856–4863. https://doi.org/10.1002/chem.201704933.

(14) Ulusoy Ghobadi, T. G.; Akhuseyin Yildiz, E.; Buyuktemiz, M.; Sadigh Akbari, S.; Topkaya, D.; İsci, Ü.; Dede, Y.; Yaglioglu, H. G.; Karadas, F. A Noble-Metal-Free Heterogeneous Photosensitizer-Relay Catalyst Triad That Catalyzes Water Oxidation under Visible Light. *Angew. Chemie* **2018**, *130* (52), 17419–17423. https://doi.org/10.1002/ange.201811570.

(15) Ghobadi, T. G. U.; Ghobadi, A.; Demirtas, M.; Buyuktemiz, M.; Ozvural, K. N.; Yildiz, E. A.; Erdem, E.; Yaglioglu, H. G.; Durgun, E.; Dede, Y.; Ozbay, E.; Karadas, F. Building an Iron Chromophore Incorporating Prussian Blue Analogue for Photoelectrochemical Water Oxidation. *Chem. - A Eur. J.* **2021**, *27* (35), 8966–8976. https://doi.org/10.1002/chem.202100654.

(16) Turhan, E. A.; Nune, S. V. K.; Ülker, E.; Şahin, U.; Dede, Y.; Karadas, F. Water Oxidation Electrocatalysis with a Cobalt-Borate-Based Hybrid System under Neutral Conditions. *Chem. - A Eur. J.* **2018**, *24* (41), 10372–10382. https://doi.org/10.1002/chem.201801412.

(17) Chemcraft - Graphical Software for Visualization of Quantum Chemistry Computations.
